# A plasma membrane localized protein phosphatase in *Toxoplasma gondii*, PPM5C, regulates attachment to host cells

**DOI:** 10.1038/s41598-019-42441-1

**Published:** 2019-04-11

**Authors:** Chunlin Yang, Malgorzata Broncel, Caia Dominicus, Emily Sampson, William J. Blakely, Moritz Treeck, Gustavo Arrizabalaga

**Affiliations:** 1 Department of Pharmacology and Toxicology, Indiana University School of Medicine, Indianapolis, IN USA; 2 Department of Microbiology and Immunology, Indiana University School of Medicine, Indianapolis, IN USA; 3 Signalling in Apicomplexan Parasites Laboratory, The Francis Crick Institute, London, United Kingdom

## Abstract

The propagation of *Toxoplasma gondii* is accomplished by repeated lytic cycles of parasite attachment to a host cell, invasion, replication within a parasitophorous vacuole, and egress from the cell. This lytic cycle is delicately regulated by calcium-dependent reversible phosphorylation of the molecular machinery that drives invasion and egress. While much progress has been made elucidating the protein kinases and substrates central to parasite propagation, little is known about the relevant protein phosphatases. In this study, we focused on the five protein phosphatases that are predicted to be membrane-associated either integrally or peripherally. We have determined that of these only PPM5C, a PP2C family member, localizes to the plasma membrane of *Toxoplasma*. Disruption of PPM5C results in a slow propagation phenotype in tissue culture. Interestingly, parasites lacking PPM5C divide and undergo egress at a normal rate, but have a deficiency in attaching to host cells. Both membrane localization and phosphatase activity are required for PPM5C’s role in attachment. Phosphoproteomic analysis show relatively few phosphorylation sites being affected by PPM5C deletion in extracellular parasites of which several are found on proteins involved in signaling cascades. This implies that PPM5C is part of a wider regulatory network important for attachment to host cells.

## Introduction

*Toxoplasma gondii*, an obligate intracellular parasite of the phylum Apicomplexa, infects warm-blooded animals including an estimated two billion people^1,2^. Infection with *Toxoplasma* occurs either by consumption of undercooked or raw meat from infected animals, ingestion of environmental oocysts that are the products of sexual reproduction in cats and are expelled in their feces, or thorough congenital transmission. In hosts other than cats, the parasite undergoes asexual reproduction and exist in two forms, a rapidly dividing tachyzoite responsible for acute infection, and a tissue cyst forming bradyzoite, which establishes a chronic infection^3^. As the acute stage is sensitive to the host’s immune response, in healthy people, *Toxoplasma* infection is usually asymptomatic. However, in the absence of a robust immune system, which is the case in those immunocompromised or immunosuppressed and in the developing fetus, *Toxoplasma* can multiply unchecked and cause life threatening disease^4–6^. Main clinical manifestations of toxoplasmosis include encephalitis and cardiomyopathy in HIV/AIDS, cancer and immunosuppressed patients and stillbirth, hydrocephalus, neurological abnormalities in congenital infections^7–9^.

The symptoms of severe toxoplasmosis are in great part the result of the tissue damage caused by repeated cycles of parasite entry into cells, replication, and lytic egress. These steps, along with attachment and motility, are exquisitely regulated by coordinated secretion from three unique organelles, micronemes, rhoptries, and dense granules^10^. Micronemes secrete proteins that are critical for motility, formation of a tight junction between the parasite and the host cell, and invasion^11^. Calcium dependent reversible phosphorylation has been shown to play a critical role in regulating microneme secretion and activation of the motility system during egress and invasion^12–14^. In particular, members of the calcium dependent protein kinase (CDPK) family, which are unique to plants and apicomplexan parasites, play critical roles during the lytic cycle of the parasite. CDPK1, for instance, has been shown to be part of a signaling pathway that leads to secretion of microneme proteins required for *Toxoplasma* motility, invasion, and egress^12^. Similarly, we have shown that CDPK3 initiates rapid egress by regulating the phosphorylation state of the motor protein myosin A (MyoA), which powers the gliding motility system required to enter and exit host cells^13^. Interestingly, many components of the parasite’s motility system, referred to as the glideosome, have been shown to be regulated by reversible phosphorylation^15,16^.

Comparative phosphoproteomic studies in *Toxoplasma* suggest that dephosphorylation also plays an important role in the parasite’s progression through its lytic cycle^15,17^. It has been reported that dephosphorylation of two residues (Ser163 and 167) of the glideosome associated protein 45 (GAP45), is required for the assembly of the MyoA-MLC1-GAP45 motor complex^18^. Nonetheless, the relevance of this dephosphorylation has been put into question by later work^16^. Dephosphorylation does appear to play a role in regulating the apical membrane antigen 1 (AMA1), which mediates host cell attachment and invasion. Dephosphorylation of the cytosolic tail of AMA1 enhances *Toxoplasma*’s invasion into host cells^19^. Thus, it is reasonable to infer that protein phosphatases play critical roles in the propagation of the parasite.

While many of the kinases that play a role in the lytic cycle have been identified, very little is known of the phosphatases that contribute to regulation of parasite propagation. *Toxoplasma* encodes more than 60 predicted protein phosphatases including 52 serine/threonine phosphatases (PSPs) and 9 tyrosine phosphatases^20^. Since most phosphorylated sites detected in lytic cycle effector proteins are primarily serine and threonine residues, phosphatases involved in their dephosphorylation are more likely to be PSPs^16^. Among these PSPs, only calcineurin, a phosphoprotein phosphatase (PPP) family PSP, has been shown to be involved in the regulation of the lytic cycle of *Toxoplasma*^21^. Specifically, calcineurin plays a role in microneme independent host cell attachment^21^.

To expand on our knowledge of phosphatases that might play a role in the regulation of the parasite’s lytic cycle, we have begun to study PSPs that would be predicted to associate with the parasite membrane. This criterion is due to the fact that the glideosome is located at the periphery of the parasite between the membrane and the inner membrane complex (IMC), a set of flattened vesicles that underlay the plasma membrane^22^. In this study, we determine that the protein phosphatase PPM5C, a PP2C family member containing both N-myristoylation and S-palmitoylation sites, localizes to the plasma membrane where it regulates attachment to host cells.

## Results

### PPM5C is localized to the plasma membrane

**Figure 1:**
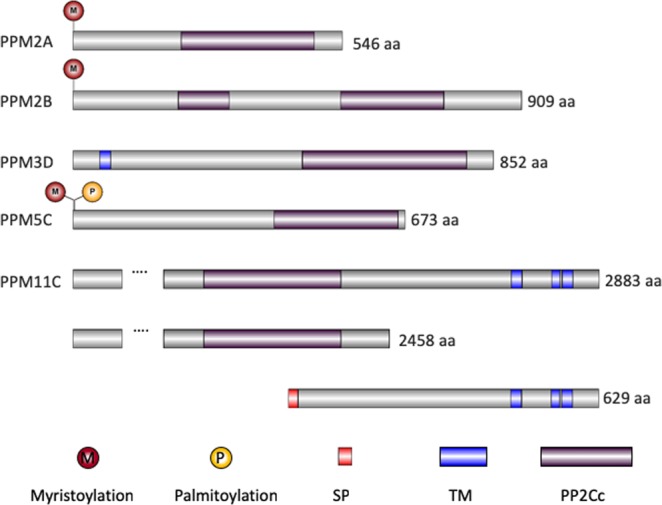
Schematic of five putative membrane associated protein phosphatases. Predicted functional domains and protein modification sites are shown for PPM2A (TGGT1_232340), PPM2B (TGGT1_267100), PPM3D (TGGT1_202610), PPM5C (TGGT1_281580), PPM11C (TGGT1_304955). Below PPM11C are the two putative proteins encoded genomic locus of TGGT1_304955. M, myristoylation; P, palmitoylation; SP, signal peptide; TM, transmembrane domain; PP2Cc, PP2C phosphatase catalytic domain.

Among the putative serine/threonine protein phosphatases in *Toxoplasma*, five have features that would predict membrane association: two containing transmembrane domains (TGGT1_202610 aka PPM3D and TGGT1_304955 aka PPM11C), two possessing predicted myristoylation sites (TGGT1_232340 aka PPM2A and TGGT1_267100 aka PPM2B) and one with both putative myristoylation and palmitoylation sites (TGGT1_281580 aka PPM5C)^20^. Relative position of these features as well as of other functional domains are shown in the schematics in Fig. 1.

**Figure 2:**
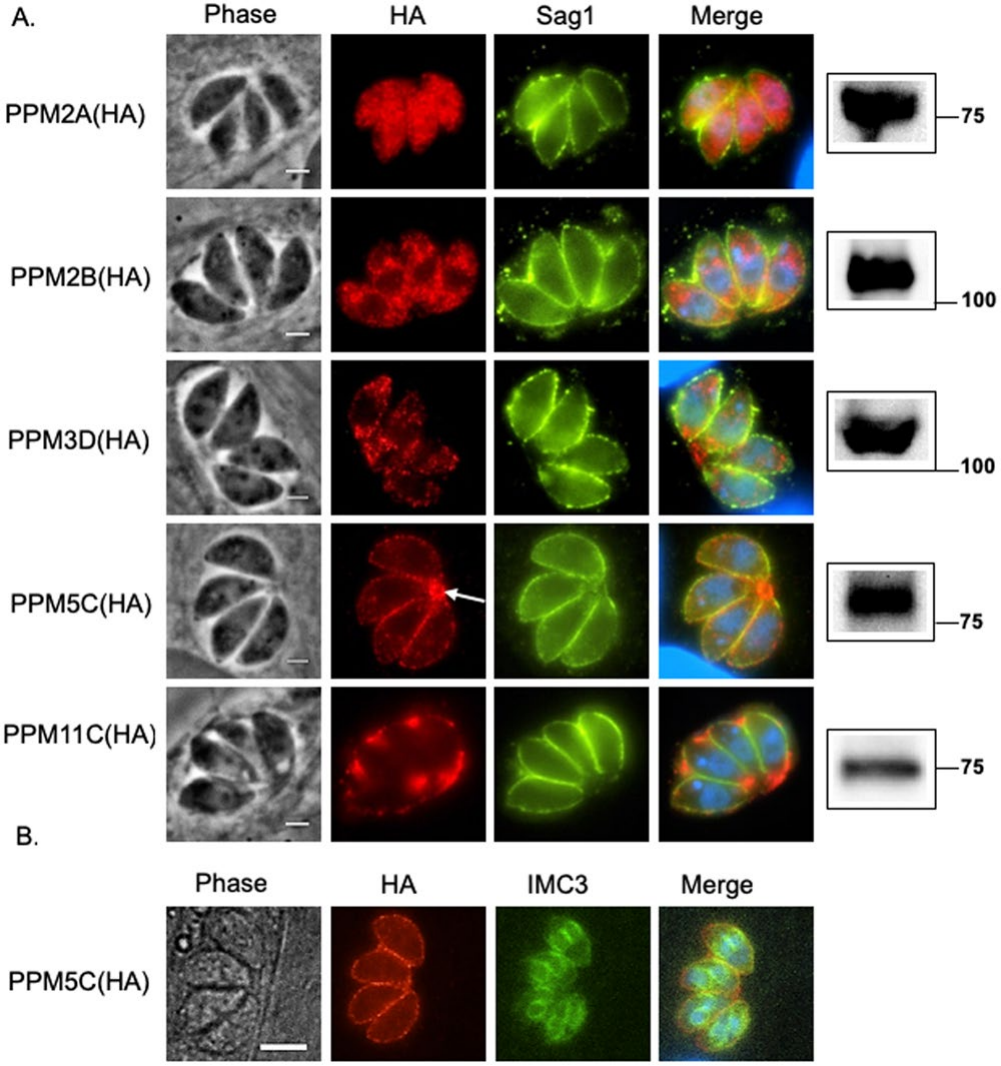
Localization of the protein phosphatases. (**A**) Intracellular parasites expressing endogenously HA tagged protein phosphatases were stained with antibodies against the HA epitope (red) and the surface protein Sag1 (green). Panels to the right show western blot of parasite extracts probed for HA. Original blots are shown in Supplemental Fig. S1. Arrow in PPM5C row points at PPM5C in the residual body. Scale bar = 2 µm. (**B)** Intracellular parasites were stained with antibodies against the HA epitope and IMC3, a component of the Inner Membrane Complex. Scale bar: 5 µm.

To investigate the localization of these phosphatases, we introduced a triple hemagglutinin (HA) epitope tag to the C-termini of the endogenous proteins. Immunofluorescence assay (IFA) of the resulting strains showed that PPM2A and PPM2B are both distributed throughout the cytosol (Fig. 2A). Interestingly, PPM2B appears to be excluded from the nucleus, while PPM2A is not. PPM3D, which has a predicted transmembrane domain, is not localized to the plasma membrane and instead it exhibits a localization pattern reminiscent of the ER. On the other hand, PPM5C appears to be localized to the periphery of the parasite, most likely to the plasma membrane (Fig. 2A). We also detected PPM5C in the residual body, a structure that arises during parasite division and contains remnants of mother cell left behind during the formation of daughter parasites (Fig. 2A, Arrow)^23^. While it is plausible that PPM5C associates with IMC, we never see it associated with the IMC of daughter cells, which can clearly be observed with IMC markers such as IMC3 (Fig. 2B).

Interestingly, we observed that PPM11C (TGGT1_304955) is secreted into the parasitophorous vacuole within which the parasite divides (Fig. 2A). Nonetheless, the protein product detected by western blot is around 75 kDa, which is significantly less than the expected 308 kDa. Careful analysis of the gene model for TGGT1_304955, suggest that it is likely incorrect and that the predicted first 5 exons, which encode the phosphatase domain, and the last 4 exons, which encode 3 C-terminal transmembrane domains, are two separate genes. Several lines of evidence support this idea. First, while the gene model predicts a 3,677 bases intron between exons 5 and 6, there is no RNA sequence evidence for such splicing event and instead there is evidence for a 500 nucleotides intron following the fifth exon which would provide an in frame stop codon (ToxoDB, RNAseq ‘evidence for introns’ dataset). Second, several data sets from chromatin precipitations suggest the presence of promoter activity within the large intron prior to what is predicted as the sixth exon. Finally, within the large intron just prior to the sixth exon there is a potential in frame start methionine that, if used would predict a 63 kDa protein with a predicted signal sequence, which is consistent with what we observe by both IFA and western blot. Thus, TGGT1_304955 is likely misannotated and it represents two different proteins, one with a protein phosphatase domain, and a secreted one with a signal sequence and transmembrane domains (Fig. 1).

### Amino acids predicted to be myristoylated or palmitoylated are both required for the plasma membrane localization of PPM5C

**Figure 3:**
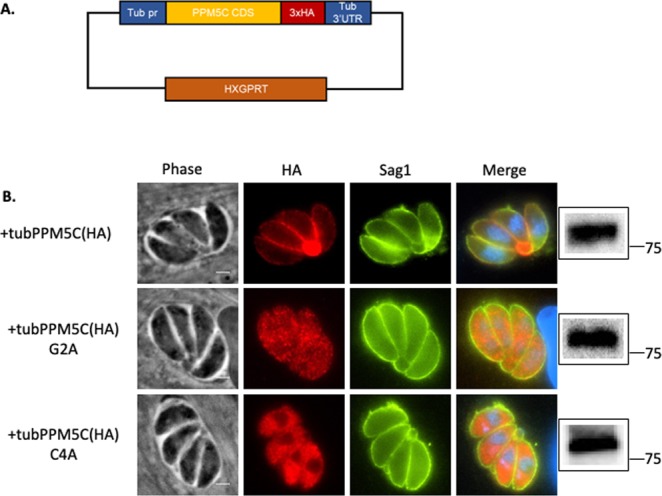
Role of amino acids predicted to be myristoylated or palmitoylated in PPM5C localization. Parasites were transfected with plasmids encoding either wild type or mutant PPM5C to determine the role of glycine 2, which is predicted to be myristoylated and cysteine 4, predicted to be palmitoylated, in localization. (**A**) Diagram of the template PPM5C expression plasmid is shown. PPM5C coding sequences are under the control of the high expressing tubulin promoter (tub pr) and include three copies of the HA epitope tag (3xHA). Plasmid also contains the selectable marker HXGPRT. (**B**) Immunofluorescence of intracellular parasites expressing exogenous wildtype PPM5C (+tubPPM5C(HA), or mutant PPM5C in which either glycine 2 or cysteine 4 are mutated for alanine (+tubPPM5C G2A and +tubPPM5C(HA) C4A). Parasites were stained for HA to detect IMC2A (red) and the surface antigen Sag1 to detect the parasite membrane (green). Western blots of protein extract of each transgenic strain probed for HA are shown on the right. Original blots are shown in Supplemental Fig. S2.

As our interest is on protein phosphatases localized to the periphery of the parasite we focused our efforts on the functional characterization of PPM5C. Two amino acids in the N-terminus of PPM5C are predicted to be post-translationally modified: a glycine at position 2 is predicted to be myristoylated (Myristoylator)^24^ and the cysteine at position 4 is predicted to be palmitoylated (CSS-Palm 4.0)^25^. To explore whether these two amino acids are required for PPM5C localization to the plasma membrane, we generated parasite strains stably expressing an exogenous copy of wildtype PPM5C or PPM5C in which either the putative myristoylation or palmitoylation sites were mutated to alanine (G2A or C4A, respectively) (Fig. 3A). Western blot showed that the exogenously expressed proteins were of the expected sizes (Fig. 3B). IFA of intracellular parasites showed that, as in the case of endogenous PPM5C, exogenously expressed PPM5C is localized to the plasma membrane and residual body (Fig. 3B). By contrast, in parasites expressing either PPM5C(G2A):3xHA or PPM5C(C4A):3xHA we detected the protein throughout the cytoplasm. The staining pattern for both mutants appears punctate, so it is plausible that both associate with membrane structures. Nonetheless, neither of the mutants is associated with the plasma membrane (Fig. 3B). These data suggest that both the second and fourth amino acids, which are putative myristoylation and palmitoylation sites, are required for plasma membrane localization of PPM5C.

### Knockout of PPM5C affects *Toxoplasma’s* rate of propagation

**Figure 4:**
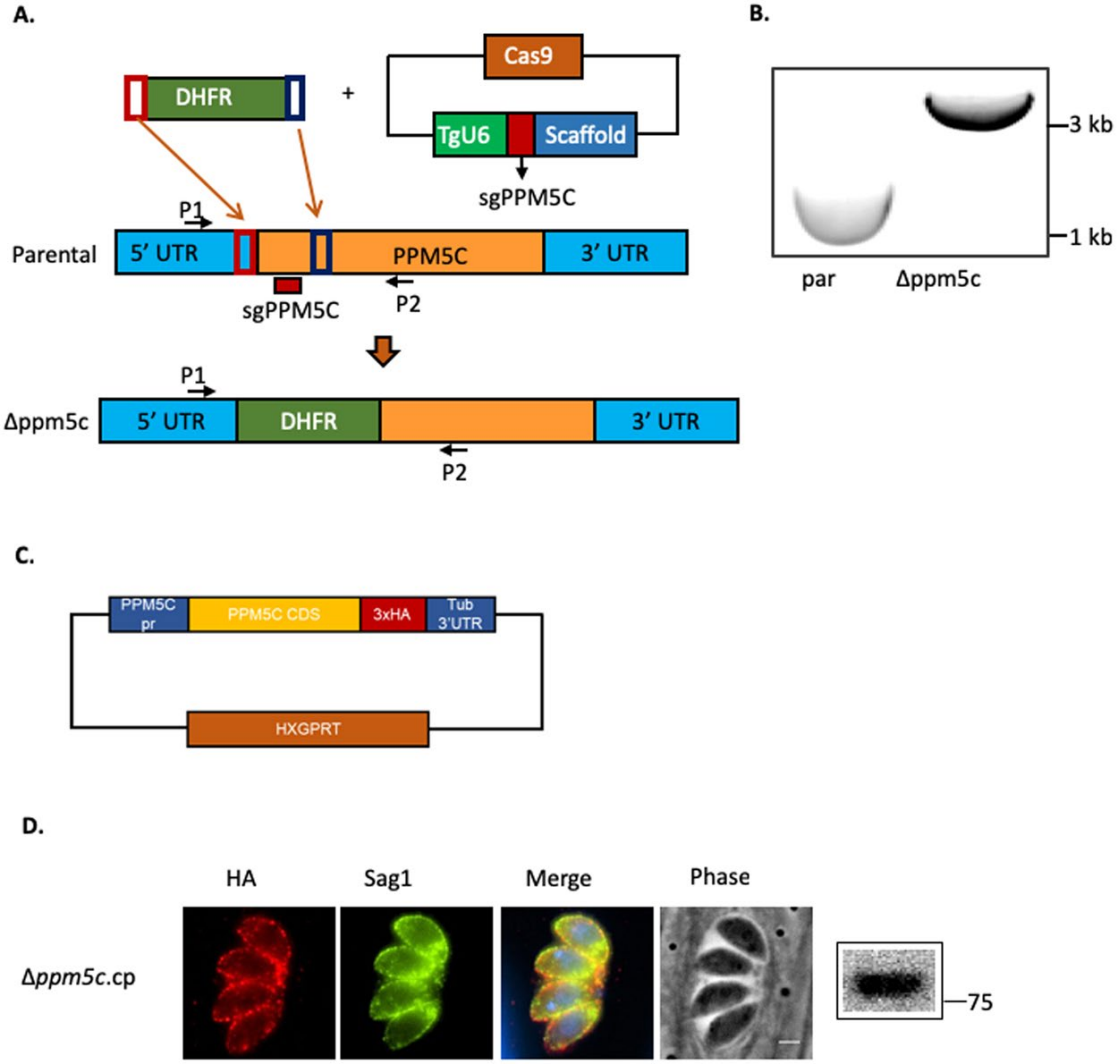
Generation and complementation of PPM5C knockout strain. (**A**) Diagram depicts strategy used for developing the PPM5C knock-out strain ∆*ppm5*. Parasites were transformed with a plasmid encoding Cas9 and a guide RNA that targets *PPM5C* first exon and a repair fragment that contains regions of homology to the PPM5C locus (red and blue boxes) flanking the selectable marker DHFR. The bottom graphic in (**A**) represents the resulting edited genome in the knock out strain. P1 and P2 are primers used to confirm disruption. (**B**) Disruption of *PPM5C* in the knockout strain was confirmed through PCR with primers P1 and P2 shown in (**A**). (**C**) Diagram depicts plasmid used to reintroduce PPM5C into the knockout strain. In this complementation plasmid, *PPM5C* is driven by its own promoter (*PPM5C* pr) and contains a triple HA epitope tag. (**D**) Expression of PPM5C in the complemented strain (∆*ppm5c*.cp) was confirmed by immunofluorescence assays and western blot. Original blots are in Supplemental Fig. S3.

To determine the function of PPM5C in *Toxoplasma*, we generated a knockout strain of *PPM5C* using a CRISPR/Cas9 driven approach (Fig. 4A)^26,27^. In brief, we transfected parasites with a CRIPSR construct, pTub-Cas9-U6-sgPPM5C, which expresses Cas9 and a guide RNA targeting the first exon of the *PPM5C* gene, and a PCR amplicon containing the dihydrofolate reductase (DHFR) selectable maker flanked by overhangs with homology to *PPM5C* sequences upstream and downstream of the Cas9 cut site (Fig. 4A). Disruption of the *PPM5C* in an established clone, referred to as ∆*ppm5c*, was confirmed by PCR (Fig. 4B) and sequencing of the resulting amplicon. To be able to ascribe any defect observed in the ∆*ppm5c* strain to the lack of PPM5C, we reintroduced the *PPM5C* cDNA with a triple HA tag driven by the *PPM5C* promoter (Fig. 4C,D). IFA of the resulting complemented strain (∆*ppm5c*.cp) showed that the reintroduced PPM5C localizes correctly to the plasma membrane (Fig. 4D).

**Figure 5:**
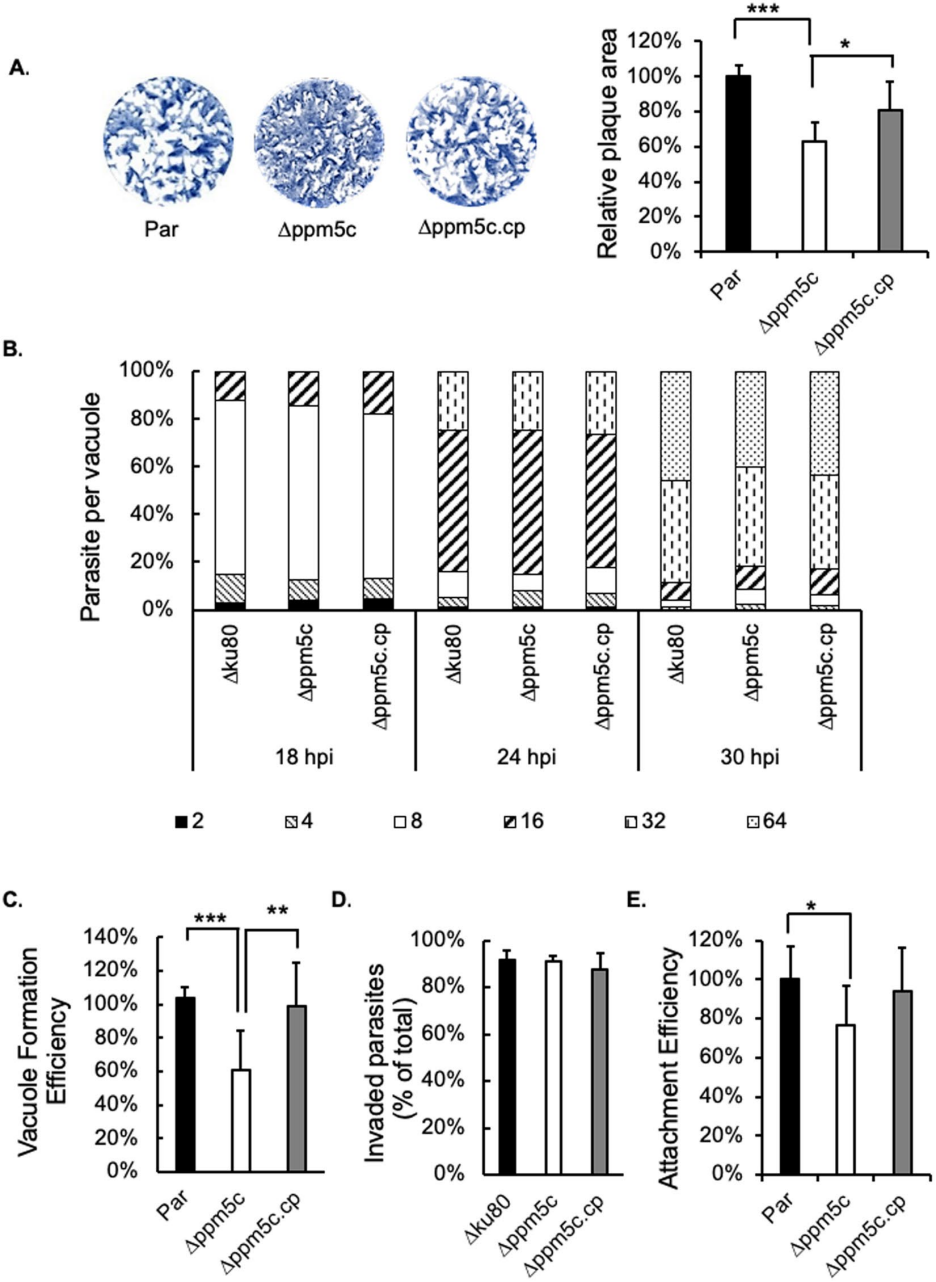
Phenotypic analysis of PPM5C knockout strain. Propagation, replication, invasion and attachment was determined for parasites of the parental (par), knockout (∆*ppm5c*) and complemented (∆*ppm5c*.*cp*) strains. (**A**) Parasites of all three strains were allowed to grow in human fibroblasts for six days before fixation and crystal violet staining to reveal plaques formed by repeating cycles of invasion, replication and egress. Representative images of plaque assays are shown as well as quantification of area cleared by plaques relative to parental strain. (**B**) Parasites were allowed to invade HFFs for 30 minutes, and infected cultures were fixed at 18, 24, and 30 hours post-infection (hpi). The proportion of vacuoles with 2, 4, 8, 16, 32 or 64 parasites was calculated for each time point and strain. (**C**) Same number of parasites of each strain were allowed to infect cells for 30 minutes and uninvaded parasites were washed out. After 24 hours cultures were fixed and the number of vacuoles in 20 randomly and blindly selected fields of view was quantitated. Data is presented normalized to the average of total number of vacuoles formed by the parental parasites. (**D**) Parasites were allowed to infect cells for 30 minutes before fixation and differential staining for parasites outside and inside of cells. Bars represent the percentage of total parasites that were inside in 10 randomly and blindly selected fields of view. (**E**) Same experiments as in (**C**) were analyzed to compare the total number of parasites both inside and outside, which represents the number of parasites that efficiently attached, in 10 randomly selected fields of view. Data is presented normalized to the average of parental parasites. For all data graphs n = 3 biological replicates × 3 experimental replicates and p-value was estimated by two tailed Student’s t-test. Error bars show standard deviations (SD). * indicates p-value < 0.05; ** indicates p-value < 0.01; *** indicates p-value < 0.001.

We examined the effect of disrupting *PPM5C* on parasite propagation by performing plaque assays of the parental, knockout, and complemented strains. This assay showed that ∆*ppm5c* parasites form significantly smaller plaques than the parental strain (Fig. 5A), suggesting knockout of *PPM5C* affects parasite propagation. This moderate plaque forming defect is consistent with the fitness score of −1.06, assigned to *PPM5C* through a genome-wide CRISPR screen^28^. Importantly, the plaque size phenotype in the knockout strain was complemented by expression of the ectopic copy of *PPM5C*, which confirms the connection between the knockout of *PPM5C* and the phenotype.

### Knockout of PPM5C causes defect in host cell attachment

Reduction in the size of plaques can be due to a disruption in any of the steps in the propagation cycle of the parasite, namely attachment, invasion, division, egress, and motility. To determine the particular step that is affected by the genetic disruption of *PPM5C*, we performed a battery of phenotypic assays. We first performed doubling assays to compare the division rate of ∆*ppm5c* to that of the parental and complement strains. Briefly, parasites were allowed to infect host cells for 30 minutes before removal of non-invaded parasites. Invaded parasites were allowed to grow for 18, 24, or 30 hours post infection at which time points cultures were fixed and the number of parasites per vacuole was enumerated for 100 vacuoles or more per experiment. This assay showed that the distribution of vacuole sizes was similar across all three strains (parental, ∆*ppm5c*, and *ppm5c*.cp) at all time points tested (Fig. 5B). This indicates that PPM5C is not needed for efficient division of the parasite.

We next focused on parasite entry into and exit from host cells. *Toxoplasma* egress is dependent on calcium fluxes and can be induced by various chemicals such as the calcium ionophore A23187^29^, zaprinast^30^, and DTT^31^. Parental, *∆ppm5c*, and *∆ppm5c*.cp parasites all showed the same egress efficiency when treated with 0.1, 0.5 or 1 µM A23187 (Fig. S4). Neither did we detect differences in egress efficiency with 500 µM or 50 µM zaprinast, and 1 µM or 5 µM DTT (data not shown). Next, we explored the ability of all three strains to enter host cells and establish parasitophorous vacuoles. Briefly, the same number of parasites of each strain were added to host cell monolayers and allowed to invade for 30 minutes before removal of free extracellular parasites. After 24 hours of incubation, at which time points parasites remain within the initial parasitophorous vacuole, cultures were fixed, and the number of parasite vacuoles were counted (Fig. 5C). The number of vacuoles formed by ∆*ppm5c* parasites was approximately 40% less than those formed by either the parental or complemented strains (Fig. 5C). This result suggests that the propagation phenotype observed with the *∆ppm5c* parasites is likely due to a defect in one of the steps involved in parasite entry into the host cell.

To more precisely analyze the effects of PPM5C disruption on invasion, parasites were allowed to invade for just 30 minutes. After washing off unattached parasites, cultures were fixed and processed with a differential staining method that distinguishes parasites that are outside the host cell from those that are inside^32^. The ratio of parasites that are inside over the total number of parasites in the culture (both inside and outside) represents the percentage of parasites that have invaded cells once attached. Comparison of this ratio across the three strains shows equivalent efficiency to invade host cells (Fig. 5D). Interestingly, we observed that although the same number of parasites of each strain was used in this assay, the total number of parasites detected was consistently 20–25% less for ∆*ppm5c* than either the parental or complemented strains (Fig. 5E). As ∆*ppm5c* parasites can invade normally once attached (Fig. 5C) the reduction in number of parasites in relation to the other two strains indicates a defect in attachment. These results indicate that inefficient attachment might account for the propagation defect observed in the PPM5C knockout parasites.

### Microneme secretion is not affected by the knockout of PPM5C

**Figure 6:**
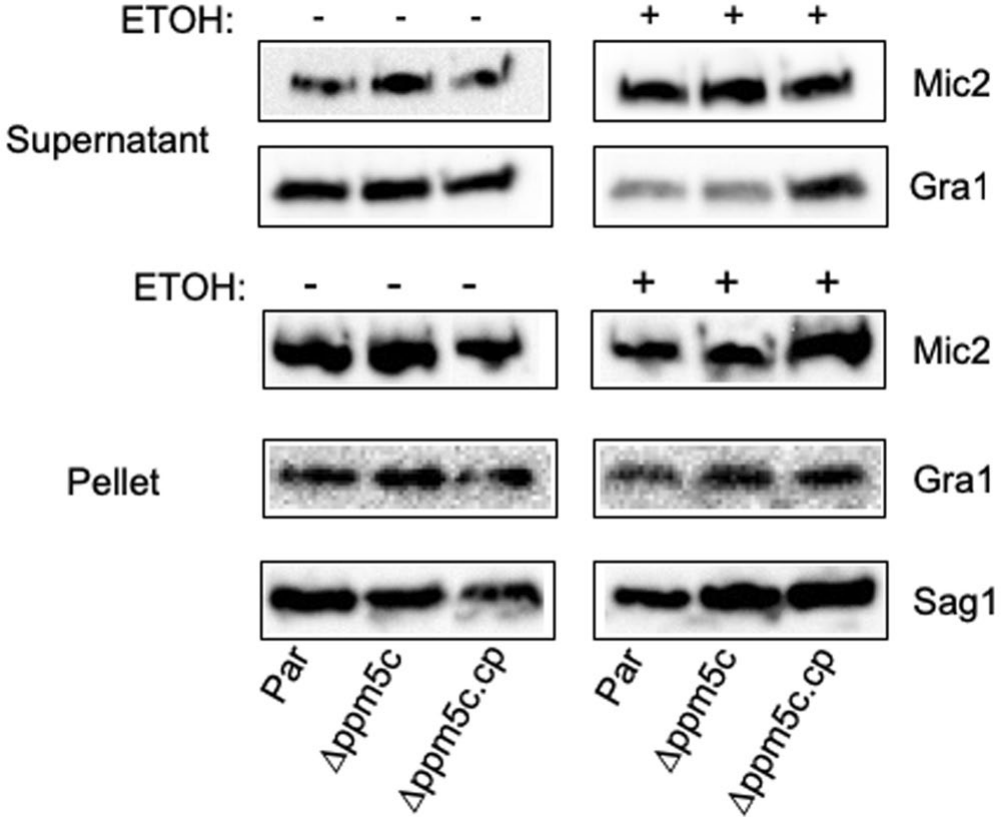
Microneme secretion in PPM5C knockout strain. Extracellular parasites of the parental (par), knockout (∆*ppm5c*) and complemented (∆*ppm5c*.*cp*) strains were incubated for 10 minutes with or without ethanol and spun down. Supernatant, which contains secreted antigens, and pellet, which contain whole parasites, were processed for western blots. Secreted microneme protein Mic2 and secreted dense granule protein Gra1 was detected in the supernatant (top two rows). In the absence of ethanol constitutive and baseline secretion is detected, while ethanol induces calcium dependent microneme secretion. To confirm equal number of parasites protein extract from the parasite pellet was probed with the surface antigen Sag1. Additionally, we probed for Mic2 and Gra1 in extracts from the parasite pellets to confirm equal levels of the proteins across strains tested. All original blots are shown in Supplemental Fig. S5.

The establishment of a tight interaction between the parasite and host cell is mediated by several proteins secreted from micronemes in a calcium dependent manner^12,33–35^. To assess whether the attachment phenotype of the ∆*ppm5c* parasites is the consequence of insufficient microneme secretion, we compared the levels of secreted MIC2 between parental, ∆p*pm5c*, and ∆*ppm5c*.cp strains (Fig. 6). We detected no difference in the amount of MIC2 constitutively secreted by the three strains. In addition, we also compared calcium induced microneme secretion by incubating parasites with ethanol and detected no difference in the amount of secreted MIC2 between the three strains (Fig. 6). There was also no difference in constitutive secretion from a second set of secretory organelles, the dense granules, which was assessed by probing for Gra1 (Fig. 6). In conjunction, these data suggest that the role of PPM5C in attachment is likely independent of microneme secretion.

### Localization and activity are important for PPM5C’s role in attachment

**Figure 7:**
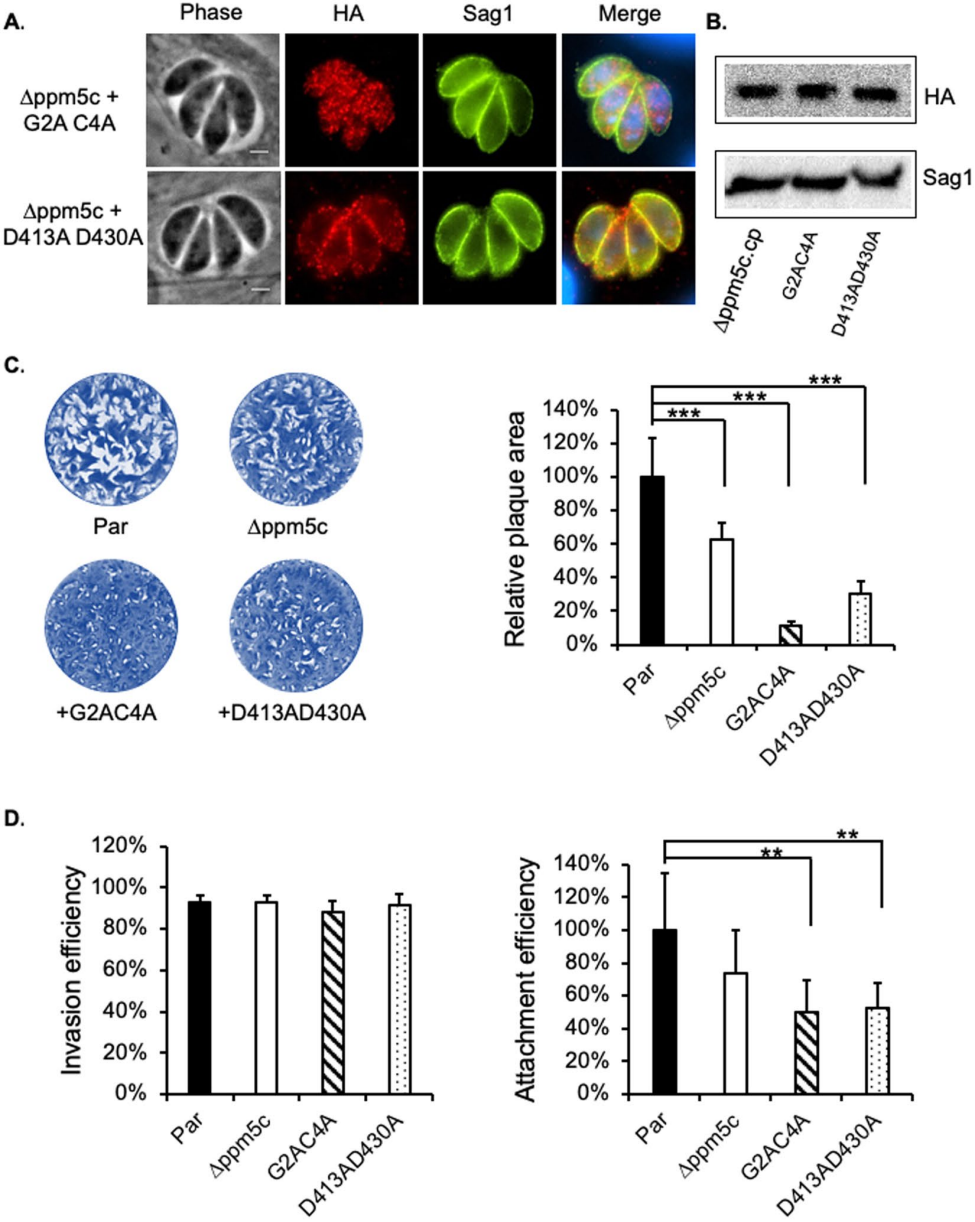
Role of membrane localization and phosphatase activity in PPM5C function. The knockout strain ∆*ppm5c* was transfected with mutant versions of the complementation plasmid shown in Fig. 4C. In mutant G2A C4A, the predicted myristoylated and palmitoylated sites are changed for alanine, which mislocalize the protein to cytoplasm. In the D413A D430A mutant, two ion binding aspartate sites D413 and D430, which are required for phosphatase activity, are changed for alanine. (**A**) Intracellular parasites of the knockout strain complemented with either the G2A C4A or the D413A D430A mutant PPM5C were stained for HA to determine the localization exogenous mutant PPM5C. As expected, G2A C4A is mislocalized to the cytoplasm and D413A D430A correctly associates with the plasma membrane of the parasite. (**B**) Equal expression levels of mutant and wild type PPM5C in the complemented strains was verified by western blot probed with HA antibodies using Sag1 as a loading control. Original blots are in Supplemental Fig. S6. (**C**) Representative images of plaque assay of the parental (par), knockout (∆*ppm5c*) and mutant complemented (G2A C4A or the D413A D430A) strains are shown. Graph on the left is the quantification of the plaque area relative to parental calculated as in Fig. 5B. (**D**) Invasion (left) and attachment (right) efficiency was determined for all strains as described for Fig. 5D,E. For all data n = 3 biological replicates × 3 experimental replicates and p-value was estimated by two tailed Student’s t-test. Error bars show standard deviations (SD). * indicates p-value < 0.05; ** indicates p-value < 0.01; *** indicates p-value < 0.001.

We have confirmed the necessity of the amino acids predicted to be myristoylated and palmitoylated for plasma membrane localization of PPM5C. To determine whether this localization was needed for its function as it relates to attachment, we tested whether PPM5C containing mutations of both the putative myristoylation and palmitoylation sites (G2A C4A) was able to complement the knockout strain. As expected, PPM5C G2A C4A did not localize to the membrane (Fig. 7A). Importantly, while expression of PPM5C complemented the plaquing and attachment phenotypes observed in the *∆ppm5c* strain (Fig. 5), mislocalized PPM5C G2A C4A was unable to complement either the plaquing (Fig. 7C) or the attachment (Fig. 7D) phenotypes. The inability to complement these phenotypes is not due to differences in protein levels as the mutant and wildtype PPM5C expressed at similar levels based on western blot analysis (Fig. 7B). We also used the same complementation approach to test whether the role of PPM5C in host cell attachment relies on its phosphatase activity. To this end, we complemented ∆*ppm5c* with PPM5C in which the two conserved metal binding aspartic acids, D413 and D430, were mutated to alanine. This mutant version (PPM5C D413A D430A) localizes correctly to the membrane (Fig. 7A) and expresses at similar levels as exogenous wildtype PPM5C (Fig. 7B). Nonetheless, in contrast to wildtype PPM5C, the D413A D430A mutant failed to rescue either the plaquing (Fig. 7C) or attachment phenotypes (Fig. 7D). Interestingly, we observed that, in the ∆*ppm5c* strain complemented with either the localization or activity mutant, the plaquing and attachment phenotypes were more severe than those observed in the knockout strain (Fig. 7C,D), suggesting that over expression of mutant PPM5C exerts a dominant negative effect. In conjunction, these results indicate that PPM5C phosphatase activity is required at the periphery of the parasite for efficient attachment and propagation.

### Identification of PPM5C interactors and proximal proteins

To identify potential substrates of PPM5C, we performed biotinylation by antibody recognition (BAR), a method for proximity labeling that allows for mapping of proteins that are adjacent to or interact with a protein of interest^36^. Given the relatively low expression of endogenous PPM5C, we generated a parasite strain that overexpresses PPM5C. To this end, we complemented Δ*ppm5c* with the *PPM5C* coding sequence driven by a *Toxoplasma* tubulin promoter. The obtained strain *Δppm5c*.oe showed that overexpressed PPM5C localized to the plasma membrane and complemented *Δppm5c* phenotypes confirming that it is functional (Fig. S7). As a control we used the parental Δ*ku80*, which does not express HA. To label the proteins in proximity to PPM5C by biotinylation, *Δppm5c*.oe intracellular parasites were fixed, permeabilized, and incubated with rabbit anti-HA antibodies and horseradish peroxidase (HRP) conjugated anti-rabbit IgG. Cultures were then exposed to biotin phenol and hydrogen peroxide to induce biotinylation of proteins in proximity to the HRP bound PPM5C. As a validation of the method and controls we first performed a small-scale experiment on parasites grown on coverslips and detected the labeled proteins by IFA with Alexa-Fluor conjugated Streptavidin (Fig. S8). As expected, when using the PPM5C.3xHA expressing strain the signal was enriched at the periphery of the parasites (Fig. S8). In contrast, when we performed the same experiment with the PPM5C(G2A).3xHA expressing strain in which PPM5C is mislocalized, the signal was distributed throughout the cytoplasm (Fig. S8).

Based on this result we performed a large scale biotinylation trial and purified biotinylated proteins using streptavidin conjugated magnetic beads and identified them using mass spectrometry. Experiments and controls were performed in duplicate. We applied the following criteria to develop a list of putative PPM5C interactors or proximal proteins: peptides in both experimental duplicates, no less than 10 peptides between the two experimental BAR assays and fold change in total number of peptides of >2 between HA vs no HA expressing strains in both experiments and of >2.5 for total number of peptides. In this manner, we generated a list of 277 proteins (Supplemental Dataset S1). A significant number of these (36 proteins) are known components of the parasite pellicle, which is consistent with the localization of PPM5C to the plasma membrane of the parasite (Supplemental Dataset S1, gray cells). These include all glideosome associated proteins, 14 IMC proteins, as well as signaling proteins that localize to the periphery such as TgCDPK1 and TgCDPK2A. To further refine this list of putative near neighbors and interactors we performed the same experiment with the strain expressing the mislocalization mutant PPM5C(G2A):3xHA. The rationale for using the mislocalized PPM5C, is that as it does not rescue the phenotype of the *ppm5c* knockout parasites. We performed these control experiment in duplicate. When using a filter of a ≥2.5 fold difference in total peptides from experiments with the wildtype PPM5C to total peptides with mislocalized mutant we reduce our list of putative neighbors/interactors to 219 (Supplemental Dataset S1, PPM5C interactome vs mutant tab). The proteins eliminated by this filter are predominantly ribosomal proteins, which are common contaminants. Table 1 shows the 22 proteins for which we detected peptides in the experimental BARs but not in any of the controls, which represents the most stringent of criteria. Notable among these proteins are the glideosome-associated protein with multiple-membrane spans GAPM2B^37^, members of the thioredoxin-like proteins that associate with the cortical microtubules, TLAP3 and TLAP4^38^ and the IMC proteins IMC13 and ISC6^22,39^.

**Table 1:** Proteins identified through biotinylation by antibody recognition (BAR) from the PPM5C-HA expressing parasites but not from any of the controls.

ID	Annotation
TGGT1_202870	SAP domain-containing protein
**TGGT1_201760**	TLAP4 (Thioredoxin-like associated protein 4)
TGGT1_269885B	rhoptry metalloprotease toxolysin TLN1
TGGT1_275650	hypothetical protein
TGGT1_227030	hypothetical protein
**TGGT1_225690**	Apical Cap Protein 7 AC7
TGGT1_231440	LsmAD domain-containing protein
TGGT1_249780	hypothetical protein
TGGT1_294200	glucose-6-phosphate 1-dehydrogenase
TGGT1_305050	putative calmodulin
TGGT1_247000	tetratricopeptide repeat-containing protein
TGGT1_266080	hypothetical protein
TGGT1_290700	hypothetical protein
**TGGT1_206690**	glideosome-associated protein with multiple-membrane spans GAPM2B
**TGGT1_253470**	alveolin domain containing intermediate filament IMC13
TGGT1_312530	splicing factor, CC1 family protein
TGGT1_217510	hypothetical protein
TGGT1_278530	putative multiprotein bridging factor type 1 family transcriptional co-activator
TGGT1_314070	hypothetical protein
TGGT1_200360	hypothetical protein
**TGGT1_235380**	TLAP3 (Thioredoxin-like associated protein 3), Apical Cap Protein 5 (AC5)
**TGGT1_267620**	IMC Sutures Component 6 (ISC6)
TGGT1_311090	ubiquitin carboxyl-terminal hydrolase
TGGT1_219140	EF-1 guanine nucleotide exchange domain-containing protein
TGGT1_318410	putative TCP-1 chaperonin

Proteins known to be part of the parasite pellicle are highlighted in bold.

As a complementary approach to identifying interactors we performed a co-immunoprecipitation (co-IP) assay. As the interactions between a phosphatase and its substrates are likely temporary, we used cross-linking with 1% formaldehyde prior to immunoprecipitation as to stabilize protein-protein interactions. Co-immunoprecipitation was performed by using mouse anti-HA magnetic beads to capture PPM5C-HA and interacting proteins. Criteria to evaluate MS results were more than 2 peptides for the experimental sample and three times more peptides in the experimental sample than in the control IP performed with IgG conjugated magnetic beads. Applying this filter to the resulting MS data resulted in a list of 26 proteins (Table 2), which included three glideosome proteins (GAP50, MyoA and MLC1), five IMC proteins (IMC1, IMC3, IMC4, IMC10 and IMC12), and one transmembrane microneme protein MIC6. All these proteins were also identified by the BAR approach. Thus, mapping of PPM5C protein-protein interactions suggests association with members of the glideosome and of the parasite pellicle.

**Table 2:** List of putative PPM5C interactors and substrates identified by Co-IP.

ID	Function annotation	Peptide Spectrum	
		IgG	HA
TGGT1_266960	beta-tubulin	0	22
TGGT1_221620	putative beta-tubulin	0	19
**TGGT1_316400B**	alpha tubulin TUBA1	0	16
**TGGT1_219320**	acid phosphatase GAP50	0	8
**TGGT1_235470**	myosin A	0	8
TGGT1_231630	alveolin domain containing intermediate filament IMC4	0	5
**TGGT1_231640**	alveolin domain containing intermediate filament IMC1	0	5
**TGGT1_248700**	alveolin domain containing intermediate filament IMC12	0	4
**TGGT1_311240**	putative DnaJ family chaperone	0	4
**TGGT1_216000**	alveolin domain containing intermediate filament IMC3	0	3
**TGGT1_230210**	alveolin domain containing intermediate filament IMC10	0	3
**TGGT1_232410**	PDI family protein	0	3
TGGT1_249900	putative adenine nucleotide translocator	0	3
**TGGT1_324600**	heat shock protein	0	3
**TGGT1_218520**	microneme protein MIC6	0	2
**TGGT1_225050**	putative adenosylhomocysteinase	0	2
**TGGT1_230160**	hypothetical protein	0	2
**TGGT1_236950**	hypothetical protein	0	2
**TGGT1_254720**	dense granule protein GRA8	0	2
**TGGT1_257680**	myosin light chain MLC1	0	2
**TGGT1_291890**	microneme protein MIC1	0	2
TGGT1_308840	SAG-related sequence SRS51 (SRS3)	0	2
TGGT1_288380	heat shock protein HSP90	1	8
TGGT1_271050	SAG-related sequence SRS34A (SAG2A)	1	5
TGGT1_233460	SAG-related sequence SRS29B (SAG1)	3	13
**TGGT1_220400**	actin depolymerizing factor ADF	1	3

The number of peptides identified by mass spectrometry in both the experimental (mouse anti-HA magnetic beads) and the control (mouse IgG magnetic beads) samples were shown to the right of each corresponding protein. The proteins **highlited** are those also identified by PPM5C biotinylation labeling.

### PPM5C influences phosphorylation state of various signaling proteins

**Figure 8:**
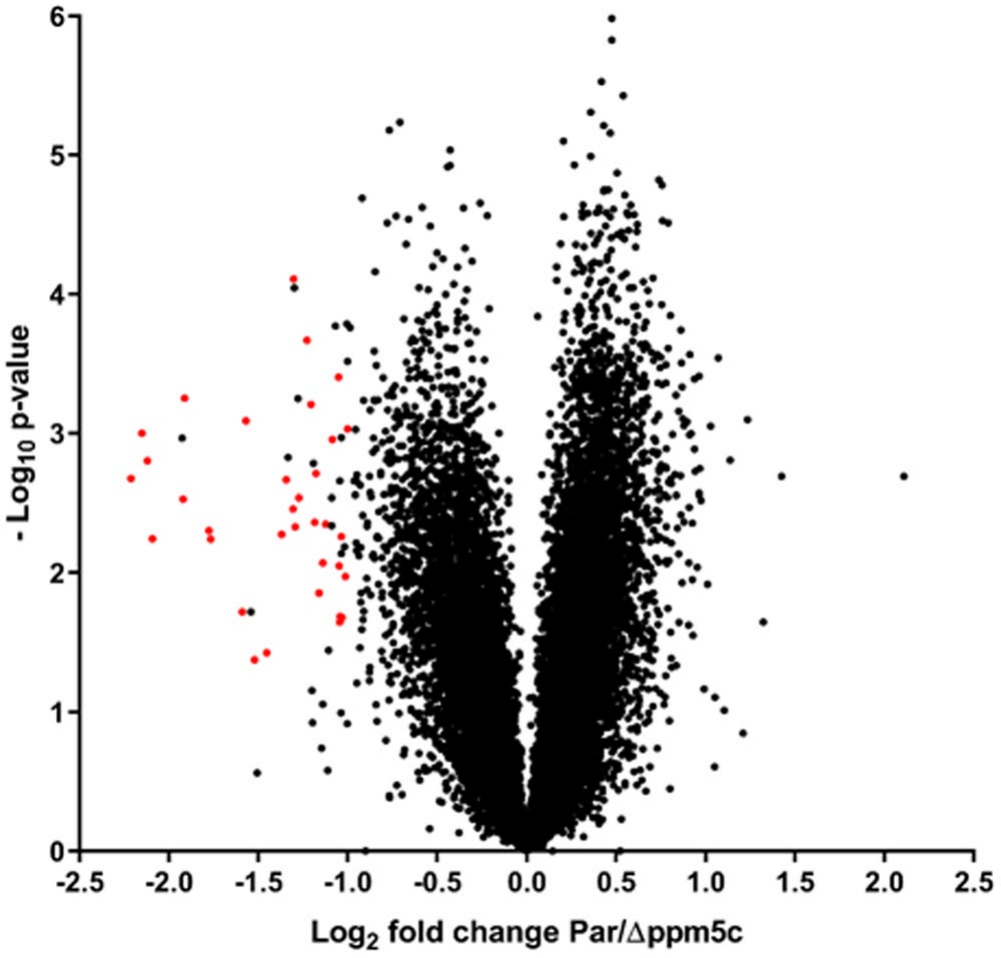
TMT quantification of change in phosphosite abundance plotted against significance of change for 18115 phosphosites in Par versus Δppm5c parasites. Phosphosites significantly more abundant upon PPM5C depletion (P-value < 0.05, log2 fold change Par/Δ*ppm5c* < −1, n = 3) and complemented upon reintroduction of PPM5C are highlighted in red. See Supplemental Dataset 2 for full data set.

To further understand the function of PPM5C, we compared phosphoproteomes of the parental, *Δppm5c*, and *Δppm5c*.cp strains using tandem mass tag (TMT) quantitative mass spectrometry based proteomics. TMT tagging allows the quantification of up to 10 conditions in parallel. To ensure conditions were consistent with our phenotypic assays, we infected HFFs, in triplicate, with parental, *Δppm5c*, and *Δppm5c*.cp for 24 hours, syringe released the parasites and left them extracellular for 30 minutes before lysis in UREA buffer and sample preparation for quantitative mass-spectrometry as detailed in materials and methods. We identified a total of 30,034 phosphosites for both human and *Toxoplasma*, and obtained quantification for 26,499 of them. *Toxoplasma* specific sites constituted 18,115 (~70%) of all quantified sites (Supplemental Dataset S2). To identify phosphosites that are significantly more abundant upon PPM5C depletion, a t-test was performed applying the following parameters: P-value < 0.05, log2 fold change parental/Δppm5c < −1 (Fig. 8). Phosphosite significance was also correlated with the PPM5C complemented sample and available proteome data to exclude phosphorylation changes originating from a non PPM5C activity and protein abundance, respectively (Supplemental Dataset S2). In total we identified 43 phosphorylation sites on 31 proteins that fulfilled these criteria (Table 3 and Supplemental Dataset S2). However, none of these proteins overlap with our biotinylation and IP data. Interestingly, one IMC protein, IMC5 appears to be more phosphorylated in the knockout strain, but this is not one of the many IMC proteins identified in the interactome. Several of the proteins identified are involved in signal transduction processes. These include kinases, a rab-GTPase, a guanylyl cyclase and a phosphatase. This suggests that PPM5C is embedded into a network of other signaling enzymes important for regulating biological processes vital for host cell attachment, some of which, such as guanylyl cyclase, also govern egress from the host-cell.

**Table 3:** Proteins identified as more phosphorylated in extracellular *∆ppm5c* parasites than in parental or complemented parasites.

ID	Annotation
TGGT1_202550	NLI interacting factor family phosphatase
TGGT1_204540	DUF367 domain-containing protein
TGGT1_213840	putative transmembrane protein
TGGT1_220090	hypothetical protein
TGGT1_220450	ribonuclease HI protein
TGGT1_224530	alveolin domain containing intermediate filament IMC5
TGGT1_228200	vacuolar (h+)-atpase g subunit protein
TGGT1_232130	hypothetical protein
TGGT1_232590	glutamate-cysteine ligase, catalytic subunit domain-containing protein
TGGT1_235000	phosphorylase family protein
TGGT1_236110	putative autophagy-related protein 3 atg3
TGGT1_243210	DUF862 domain-containing protein
TGGT1_246970	3′-5′ exonuclease domain-containing protein
TGGT1_254370	guanylyl cyclase
TGGT1_255420	hypothetical protein
TGGT1_268178	hypothetical protein
TGGT1_268430	hypothetical protein
TGGT1_285470	patched family protein
TGGT1_289880	putative transmembrane protein
TGGT1_293720	hypothetical protein
TGGT1_310530	SNF2 family N-terminal domain-containing protein
TGGT1_321340	putative membrane protein
TGGT1_221400	hypothetical protein
TGGT1_245610	hypothetical protein
TGGT1_218400	NEK kinase
TGGT1_218220	putative cell-cycle-associated protein kinase CDK
TGGT1_224270	hypothetical protein
TGGT1_235000	phosphorylase family protein
TGGT1_239580	hypothetical protein
TGGT1_269260	putative transmembrane protein
TGGT1_305920	endonuclease III family 1 protein
TGGT1_313190	Rab18/RabC-family small GTPase
TGGT1_218220	putative cell-cycle-associated protein kinase CDK
TGGT1_285470	patched family protein

## Discussion

For intracellular parasites, the ability to enter cells is central to their proliferation, propagation and, as in the case of *Toxoplasma*, pathogenesis. A key step in any invasion process is the recognition of and attachment to the cell to be infected. While for many pathogens host-cell attachment is driven by contact with specific host receptors, attachment by *Toxoplasma* appears to involve ubiquitous host factors, which is consistent with its ability to infect almost any nucleated cell. It is proposed that the initial contact between *Toxoplasma* and its host cell is mediated by the interaction of parasite glycosylphosphatidylinositol (GPI)-anchored surface antigens (SAGs) and sulfated proteoglycans on the host cell surface^40–42^. This initial attachment is followed by secretion of adhesion proteins that establish a tight interaction between the parasite and its host cell^33,41,43^. The secretion itself and the activity of some of the components of adhesion and invasion machinery are known to be tightly regulated by reversible phosphorylation^44^. Through the work described here, we have determined that PPM5C, a PP2C family protein phosphatase, is required for efficient host cell attachment by *Toxoplasma*. PPM5C is localized to the plasma membrane of the parasite possibly via lipid-anchoring served by myristoylation and palmitoylation. Complete knockout of PPM5C disrupts normal propagation of the parasite and specifically affects attachment to human cells. Importantly, we have shown that localization to the membrane and phosphatase activity are required for its function during attachment.

Disruption of attachment by either genetic or pharmacological manipulation is usually achieved by disrupting the secretion of adhesin proteins from specialized organelles known as the micronemes. Interestingly, we did not note any generalized microneme secretion defect in the PPM5C knockout strain. It is plausible that micronemal secretion is affected but not detected due to limitation in the sensitivity of our assay and the incomplete nature of the attachment defect. Nonetheless, there is precedent for attachment defects to be independent of microneme secretion. Calcineurin, a PPP family protein phosphatase is required for both *Toxoplasma* and *Plasmodium falciparum* to strongly attach to host cells before entry^21^. Knock down of the calcineurin catalytic subunit in both *Toxoplasma* and *P*. *falciparum* significantly reduced the ability of the parasites to attach to host cells, but microneme secretion levels were not influenced. Similar to what we observed with knockout of PPM5C, disruption of calcineurin does not impact any other steps of the lytic cycle other than host cell attachment. How calcineurin affects attachment at the mechanistic level and the identity of its substrates, have not been resolved. Nevertheless, our results indicate that a second phosphatase, PPM5C, influences host cell attachment independently of microneme secretion.

Transmembrane micronemal proteins, such as MIC2, MIC6, and MIC12, spanning across parasite plasma membrane with extracellular domain interact with host cell receptors and intracellular domain interact with proteins that linked to actin filaments, are central for attachment to host cells^43^. The micronemal protein AMA1, which also spans across parasites plasma membrane, is critical for attachment and invasion and has been shown to be regulated through reversible phosphorylation^19^. AMA1, along with the rhoptry proteins RON 2, 4, 5, and 8, form a ring-shaped structure, referred to as the moving junction (MJ), which spans the plasma membranes of the parasite and the host cell and serves a molecular tunnel through which the parasite enters into the host cell propelled by its motility system^45^. AMA1 uses its extracellular domain to bind to RON2, which is secreted from rhoptries and inserted into the plasma membrane of host cell where it associates with the RON4, 5, 8^27,46^. The AMA1-RON2 interaction at the moving junction generates an outside-in signal that triggers dephosphorylation of the AMA1 cytosolic tail, which is required for efficient host cell invasion^19^. Therefore, it is reasonable that the phosphorylation status of the intracellular domains of the proteins central to host cell attachment, such as AMA1, MIC2, MIC6, and MIC12, play significant roles in maximizing parasite’s attachment. Interestingly, we have identified MIC6 as a potential PPM5C interactor in both our BAR and IP analysis.

Our phosphoproteomic analysis, however, did not reveal phosphorylation sites on proteins predicted to play a role in attachment. While our phosphoproteome covers the largest amount of quantified phosphorylation sites in *Toxoplasma* so far, the amount of sites we found different between conditions was very small, but consistent. It is certainly possible that we have not investigated a perfect timepoint here, because the reversible phosphorylation involved in the regulation of attachment could happen in the short time scale when the parasites attach to host cells. In addition, other phosphatases could partially complement the phenotype, leading to only a modest effect in the phosphoproteome.

The fact that some of the proteins we identified are signaling enzymes themselves, argues that the defect in attachment of ∆ppm5c parasite may be related to the altered activity of other kinases, rather than being a direct effect. Since complementation of the KO rebalances the network, it appears to be very flexible in its response. Further work is required to identify the exact role of the identified signaling enzymes during host cell attachment. Given that some of the proteins are likely important^28^ for growth, untangling their roles in attachment versus other important biological functions will require careful analysis.

## Methods

### Parasite cultures

*Toxoplasma* tachyzoites were cultured in human foreskin fibroblasts (HFF) obtained from the American Tissue Culture Collection (ATCC) and grown in a humidified incubator at 37 °C with 5% CO_2_ in air atmosphere. The growth medium is Dulbecco’s Modified Eagle Medium (DMEM) with 10% fetal bovine serum (FBS), 2mM L-gluatamine and 50 ug/ml penicillin-streptomycin. Parasites used were of the strain RH lacking hypoxanthine-xanthine-guanine phosphoribosyl transferase (HXGPRT, RH∆*hxgprt*, referred to as ∆hx thereafter)^47^ and RH lacking HXGPRT and Ku80 (RH∆*ku80*∆*hxgprt*, referred to as ∆ku80 thereafter)^48,49^. To obtain syringed-lysed and filter-purified parasites for the different experiments, parasite infected HFF cells were scraped and passed through a 27 gauge needle to release parasites from their host cells, and then the released parasites were passed through a 3.0 uM filter to separate the free parasites in the flow through from the host cell debris.

### Establishment of transgenic parasite lines

To add an hemagglutinin (HA) epitope tag to the endogenous loci of PPM2A (TGGT1_232340), PPM2B (TGGT1_267100), PPM3D (TGGT1_202610), PPM5C(TGGT1_281580), and PPM11 (TGGT1_304955), genomic DNA fragments including the region of the target gene immediately preceding the stop codon were amplified by PCR, and cloned into the p3xHA9-LIC-DHFR vector^14^ using the In-Fusion HD Cloning Kit (Takara Bio USA, Inc.). Primers used for this and all other experiments are listed in Supplemental Table S1. The resulting plasmid was linearized within the genomic DNA fragment and transfected into ∆ku80 parasites. Transfected parasites were treated with 1 mM pyrimethamine to select for integration of constructs which include a drug resistant dihydrofolate reductase (DHFR) expression cassette. Following selection, parasites were cloned by limiting dilution, and clones were screened by immunofluorescence assays (IFAs, see below for detail).

To generate plasmids for exogenous expression of PPM5C, the *PPM5C* coding region was amplified by PCR from cDNA prepared from total RNA using the Maxima H Minus First Strand cDNA Synthesis Kit (Fisher Scientific). Total RNA was isolated from ∆ku80 parasites using the RNeasy Plus Mini Kit (QIAGEN). The resulting cDNA amplicon was cloned into the pTub-3xHA-Tub-HXGPRT vector using In-Fusion HD Cloning. This vector includes a tubulin promoter and 5′ UTR, a multicloning site to introduce the coding sequence of interest, an in-frame C-terminal triple HA epitope tag followed by stop codon and the tubulin 3′UTR, as well as a HXGPRT expression cassette for selection. The obtained plasmid, pTub-PPM5C-3xHA-Tub-HXGPRT, was then mutated at either the putative myristoylation site (G2) or the palmitoylation site (C4) to alanine using the Q5 Site-Directed Mutagenesis Kit (NEB). All resulting plasmids were linearized and transfected into the ∆hx *Toxoplasma* strain. Transfected parasites were treated with 50 mg/ml MPA and 50 mg/ml xanthine, surviving parasites were cloned by limiting dilution and the clones were screened by IFAs.

### Generation of PPM5C knockout strain

Complementary oligoes encoding the *PPM5C*-targeted guide RNA (Table S1) were annealed and incorporated into the BsaI site of the CRISPR/Cas9 vector pU6-Universal (pTub-Cas9-U6)^26^. A PCR amplicon containing the DHFR selectable marker cassette flanked by 42 base pairs of homology to the genomic region targeted by the guide RNA was amplified using pJET-DHFR (a gift from Peter Bradley) as a template. The pTub-Cas9-U6-sgPPM5C plasmid and the PCR amplicon were transfected together into ∆ku80 parasites and parasites were treated with 1 mM pyrimethamine for selection and the parasites were cloned by limiting dilution. Clones were tested by PCR and the PCR products were sequenced to confirm the desired disruption of PPM5C by insertion of DHFR.

### Complementation with PPM5C WT and mutated cDNA

The complementation plasmid was generated based on the PPM5C overexpression plasmid, pTub-PPM5C-3xHA-Tub-HXGPRT. Briefly, the tubulin promoter was replaced in the vector by a ~2000 bp DNA fragment that included the genomic sequences immediately preceding the start codon of PPM5C gene. The complementation constructs with mutant version of PPM5C were generated using Q5 Site-Directed Mutagenesis Kit (NEB). The complementation constructs were linearized and transfected into PPM5C knockout parasites along with a plasmid containing ku80, pmini-ku80 plasmid, which ensures transient expression of KU80 and allows for non-homologous end joining and random insertion^50^. The pmini-ku80 vector was generated based on the *Toxoplasma* selection plasmid pminiHXGPRT^47^. Briefly, the HXGPRT selection cassette was replaced with a ku80 expression cassette amplified from the plasmid pU6-universal-ku80 (a gift from Peter Bradley). Transfected parasites were treated with 50 mg/ml MPA and 50 mg/ml xanthine, and parasites were cloned by limiting dilution and the clones were screened by IFA.

### Immunofluorescence assay

For IFAs, parasites were added to HFFs grown on coverslips at a multiplicity of infection of 2. Cultures were grown for 18 to 24 hours prior fixation with 3.5% paraformaldehyde in phosphate‐buffered saline (PBS). After fixation, samples were blocked and permeabilized in PBS with 3% bovine serum albumin (BSA) and 0.2% Triton x-100 (TX-100). Samples were then incubated with primary antibodies in PBS/3% BSA/0.2% TX‐100 for one hour, washed five times with PBS, incubated with Alexa Fluor conjugated secondary antibodies in PBS/3% BSA for one hour. Primary antibodies used in this study included rabbit anti-HA (Cell signaling Technology) and mouse anti-Sag1, both used at 1:1000. Secondary antibodies included Alexa Fluor 594 or Alexa Fluor 488-conjugated goat anti-rabbit and goat anti mouse (Invitrogen), all used at 1:2000. After a final five washes with PBS, coverslips were mounted on slides with DAPI containing Vectashield mounting media (Vector Laboratories). The slides were inspected using a Nikon Eclipse E100080i microscope and images captured with a Hamamatsu C4742-95 charge-coupled device camera using NIS elements software.

### Western blot analysis

For western blot analysis, extracellular parasite samples were lysed in RIPA lysis buffer (50 mM Tris, 150 mM NaCl, 0.1% SDS, 0.5% sodium deoxycholate and 1% Triton X-100) containing a protease inhibitor cocktail (Cell signaling) for 1 hour. Samples were then centrifuged at maximum speed (~20,000 × g) for 10 minutes at 4 °C. Supernatant was mixed with 4X Laemmli Sample Buffer (Bio-Rad) containing 10% beta-mercaptoethanol (Thermo Scientific) and boiled for 5 minutes. Samples were then separated on a 4–20% gradient SDS‐PAGE gel (Bio‐Rad) and transferred from the gel to a nitrocellulose membrane using Trans‐Blot semidry transfer cell (Bio‐Rad). Membrane was then blocked in 5% non-fat dry milk (NFDM) in TBST (Tris-buffered saline, 0.1% Tween 20) for 1 hour, and then incubated with primary antibodies in 5% NFDM/TBST for at least 1 hour. After three washes with TBST, the membrane was incubated with anti-rabbit/mouse IgG horseradish peroxidase (HRP) in 5% NFDM/TBST for one and a half hours, washed three times with TBST, treated with SuperSignal West Pico chemiluminescent substrate (Pierce Chemical) and imaged using FluorChem E (Proteinsimple).

### Phenotypic assays

Plaque and doubling assays were performed with 12-well plates using standard methods^51^. Briefly, for the plaque assays 500 freshly syringe released and filter-purified parasites were added to confluent HFF monolaters. After six days of incubation, cultures were fixed with methanol for 5 minutes and stained with Crystal Violet. Plaques were imaged using a Proteinsimple imaging system, and the plaque areas were measured by ImageJ and the plugin ColonyArea^52^. For doubling assays around 20 thousand freshly syringe-released and filter-purified parasites were allowed to invade HFFs for 30 minutes. Cultures were washed with DMEM six times to remove uninvaded parasites and incubated for 18, 24 and 30 hours before fixation with methanol and staining with Hema3 Manual Staining System (Fisher Scientific). For each sample, the number of parasites per vacuole was recorded for 50 randomly selected vacuoles.

For egress assays, parasites were allowed to invade host cells for 24 hours, cultures were washed with PBS one time, and then incubated with 0.1, 0.5 or 1 µM A23187, 500 µM or 50 µM zaprinast, and 1 mM or 5 mM Dithiothreitol (DTT) in Hank’s Balanced Salt Solution (HBSS) for different time lengths at 37 °C. After the treatment, cultures were fixed with methanol for five minutes and stained with Hema3 Manual Staining System to count the number of intact and lysed vacuoles.

To perform vacuole formation assays, half a million of freshly syringe-released and filter-purified parasites were allowed to invade HFFs for 30 minutes infection. Unattached and uninvaded parasites were removed by three washes with DMEM and the cultures were incubated for 24 hours before fixation and staining with Hema3 Manual Staining System. For each sample, ten randomly selected field of views were scored for number of vacuoles. For the red/green invasion assays, HFFs grown in cover slips were infected with 2.5 million parasites for 30 minutes followed by three washes with DMEM, and fixation with 4% methanol-free paraformaldehyde for 15 minutes and incubated with mouse anti-Sag1 (1:2000) antibody in 3% BSA/PBS for 40 minutes. After three washes with PBS to eliminate unbound antibodies, samples were permeabilized and treated as described above for IFAs using rabbit anti-Mic5 (1:2000) antibody primary antibody goat anti-rabbit/mouse Alexa Fluor 594/488 (1: 2000). For each cover slip, ten random views were selected for counting extracellular (attached) and intracellular (invaded) parasites.

### Microneme secretion assay

Freshly lysed parasites were harvested, filter-purified and centrifugated at 1000 × g for 10 minutes. Invasion medium (DMEM/20 mM HEPES/3% FBS) was used to resuspend parasites at a concentration of 10^9^ parasites/ml. Two aliquots of 100 µl of the parasite suspension were used for the secretion assay for each sample, one for ethanol stimulation, and the other for natural secretion without stimulation. For ethanol stimulation, 2% ethanol was added. Samples were incubated at 37 °C for 10 minutes before centrifugation at 1000 × g for 3 minutes. After centrifugation, 80 µl of supernatant and the pellets were collected. Supernatant was centrifugated again and 60 µl of the new supernatant for western blotting. Pellets were lysed with RIPA buffer and prepared for western blotting as described above.

### Immunoprecipitation assay with cross linking

Freshly lysed parasites were harvested, filter-purified and centrifugated at 1000 × g for 10 minutes. Parasites were resuspended in 1 ml of PBS with 1% formaldehyde and incubate at room temperature with gentle agitation for 10 minutes. Parasites were centrifugated and resuspended with 0.125 M glycine solution in PBS and incubated at room temperature for 5 minutes to stop the cross-linking reaction. Parasites were centrifugated and washed with PBS one time and then resuspended with IP lysis buffer (25 Mm Tris-HCl pH 7.4, 150 mM NaCl, 1 mM EDTA, 1% NP-40 and 5% glycerol) and incubated at room temperature for 1 hour with rocking. The lysate was then centrifugated at maximum speed (~ 20,000 xg) for 10 minutes. The supernatant was collected and precleaned by incubation with 25 ul of mouse IgG magnetic beads (Cell signaling) for 1 hour at room temperature. The unbound lysate was isolated from the IgG beads by using a magnetic bead rack and were incubated with 25 ul of anti-HA magnetic beads (Fisher Scientific) for 1 hour at room temperature. Both mouse IgG (used as a control) and anti-HA magnetic beads were washed with IP lysis buffer for three times and with PBS for another three times.

For mass spectrometry analysis, samples were submitted to the Indiana University School of Medicine Proteomics Core facility for protein identification by mass spectrometry as described before^53^.

### Biotinylation by antibody recognition

Methods for BAR were adapted from a recently published method^36^ for use in *Toxoplasma*. Freshly lysed parasites were harvested, filter-purified and centrifugated at 1000 × g for 10 minutes. Parasites were resuspended with 1 ml of PBST (PBS with 0.1% Tween 20) with 4% paraformaldehyde for 20 minutes and quenched with 0.125 M glycine solution in PBS for 5 minutes. Parasites were washed with PBST and permeabilized with PBS with 0.5% triton X-100 for 10 minutes. Parasites were then washed with PBST and incubated with PBS with 0.3% hydrogen peroxide for 10 minutes to deactivate endogenous peroxidase activities. After one wash with PBST, parasites were blocked with 1% BSA in PBST for 2 h, and then stained with primary antibody in 1% BSA/PBST at the concentration of 1:1000 overnight at 4 °C. After five washes with PBST, parasites were incubated with secondary HRP antibody at 1:1000 in 1% BAS/PBST for 1 h. After five washes with PBST, parasites were resuspended with 100 µl of PBS containing 1 mM biotin phenol (Iris Biotech GmbH) for 10 minutes, and then mixed with 100 ul of PBS containing 1 mM biotin phenol and 2 mM hydrogen peroxide for biotinylation reactions. The reaction was allowed to continue for five minutes and then terminated by addition of 1 ml of 500 mM sodium ascorbate for five minutes. After two washes, parasites were resuspended with 300 ul lysis buffer (PBST with 1.5% SDS and 1% sodium deoxycholate) and heated to 99 °C for 1 hour. The lysate was added with 300 ul of PBS and then centrifugated at the highest speed (~20,000 × g) for 10 minutes. The isolated supernatant was incubated with 100 ul of streptavidin beads (Fisher Scientific) overnight at 4 °C. The beads were washed with PBST for two times, 1 M NaCl in PBST for two times and PBS for three times. The beads were then processed for Mass Spectrometry as described above.

### Phosphoproteome analysis

All reagents were obtained from Sigma-Aldrich unless specified otherwise. Parental (Par), Δ*ppm5c* and Δ*ppm5c*.cp *Toxoplasma* parasites were cultured in DMEM supplemented with 10% FBS. 24 hours prior to cell lysis, HFFs were infected (MOI = 5) with Par, Δ*ppm5c* and Δ*ppm5c*.cp parasites, each in biological triplicate. Parasites were syringe lysed in DMEM and left extracellular for 30 minutes at room temperature (RT). Parasites were then collected by centrifugation (1900 rpm, 10 minutes, RT) and washed once with PBS. Subsequent lysis was performed in ice cold 8 M urea, 75 mM NaCl, 50 mM Tris, pH 8.2, supplemented with protease (complete mini, Roche) and phosphatase (Phos Stop, Roche) inhibitors. Lysis was followed by sonication to reduce sample viscosity (30% duty cycle, 3 × 30 seconds bursts, on ice). Protein concentration was measured using a BCA protein assay kit (Thermo Fisher Scientific). Lysates (0.7 mg each) were subsequently reduced with 5 mM DTT for 30 minutes at 56 °C and alkylated in the dark with 14 mM iodoacetamide for 30 minutes at RT. Following iodoacetamide quenching with 5 mM DTT for 15 minutes in the dark, lysates were diluted with 50 mM ammonium bicarbonate to <4 M urea, and digested with LysC (Promega) for 2–3 hours at 37 °C. Lysates were further diluted with 50 mM ammonium bicarbonate to <2 M urea and digested with trypsin (Promega) overnight at 37 °C. After digestion, samples were acidified with trifluoroacetic acid (TFA) (Thermo Fisher Scientific) to a final concentration of 1% (v/v). All insoluble material was removed by centrifugation and the supernatant was desalted on Sep-Pak C_18_ cartridges (Waters).

For TMT labelling samples were dissolved at 1 mg/ml in 50 mM Na-Hepes, pH 8.5 and 30% acetonitrile (v/v) and labelled with respective TMT reagents (Thermo Fisher Scientific, 1.6 mg reagent/0.7 mg sample) for 1 hour at RT. Labelling was then quenched with 0.3% hydroxylamine for 15 minutes at RT and samples acidified (pH~2) with formic acid. After verification of labelling efficiency via mass spectrometry, the lysates were mixed in a 1:1 ratio, vacuum dried and desalted on Sep-Pak C_18_ cartridges.

For phosphopeptide enrichment desalted and vacuum dried samples were solubilised in 1 ml of loading buffer (80% acetonitrile, 5% TFA, 1 M glycolic acid) and mixed with 5 mg of TiO_2_ beads (Titansphere, 5 µm GL Sciences Japan). Samples were incubated for 10 minutes with agitation, followed by a 1 minute 2000 × g spin to pellet the beads. The supernatant was removed and used for a second round of enrichment as explained below. Beads were washed with 150 μl of loading buffer followed by two additional washes, the first with 150 μl 80% acetonitrile, 1% TFA and the second with 150 μl 10% acetonitrile, 0.2% TFA. After each wash, beads were pelleted by centrifugation (1 minute at 2000 × g) and the supernatant discarded. Beads were dried in a vacuum centrifuge for 30 minutes followed by two elution steps at high pH. For the first elution step, beads were mixed with 100 μl of 1% ammonium hydroxide (v/v) and for the second elution step with 100 µl of 5% ammonium hydroxide (v/v). Each time beads were incubated for 10 minutes with agitation and pelleted at 2000 × g for 1 minute. The two elutions were removed following each spin, and subsequently pooled together before undergoing vacuum drying. The supernatant from the TiO_2_ enrichment was desalted on Sep-Pak and the High Select Fe-NTA phosphopeptide enrichment kit (Thermo Fisher Scientific) was used according to manufacturer’s instructions for a second round of enrichment. The supernatant containing all non-phosphorylated peptides (total proteome) was removed and stored at −80 °C.

Combined TiO_2_ and Fe-NTA phosphopeptide eluates, as well as an aliquot of the total proteome sample (100 μg) were fractionated using the Pierce High pH Reversed-Phase kit (Thermo Fisher Scientific) according to manufacturer’s instructions. Resulting fractions were taken to dryness by vacuum centrifugation and further desalted on a stage tip using Empore C18 discs (3 M). Briefly, each stage tip was packed with one C18 disc, conditioned with 100 µl of 100% methanol, followed by 200 µl of 1% TFA. The sample was loaded in 100 μl of 1% TFA, washed 3 times with 200 µl of 1% TFA and eluted with 50 µl of 50% acetonitrile, 5% TFA. The desalted peptides were vacuum dried in preparation for LC-MS/MS analysis.

For LC-MS/MS samples were resuspended in 0.1% TFA and loaded on a 50 cm Easy Spray PepMap column (75 μm inner diameter, 2 μm particle size, Thermo Fisher Scientific) equipped with an integrated electrospray emitter. Reverse phase chromatography was performed using the RSLC nano U3000 (Thermo Fisher Scientific) with a binary buffer system (solvent A: 0.1% formic acid, 5% DMSO; solvent B: 80% acetonitrile, 0.1% formic acid, 5% DMSO) at a flow rate of 250 nl/minute. The samples were run on a linear gradient of 5–60% B in 150 minutes with a total run time of 180 minutes including column conditioning. The nanoLC was coupled to an Orbitrap Fusion Lumos mass spectrometer using an EasySpray nano source (Thermo Fisher Scientific). The Orbitrap Fusion Lumos was operated in data-dependent mode acquiring HCD MS/MS scans (R = 50,000) after an MS1 scan (R = 120, 000) on the 10 most abundant ions using MS1 target of 4 × 10^5^ ions, and MS2 target of 2 × 10^5^ ions (phospho) and 5 × 10^4^ ions (proteome). The maximum ion injection time utilized for MS2 scans was 86 ms, the HCD normalized collision energy was set at 38 and the dynamic exclusion was set at 60 seconds. Acquired raw data files were processed with MaxQuant^54^ (version 1.5.2.8) and peptides were identified from the MS/MS spectra searched against *T*. *gondii* (ToxoDB, 2018) and *Homo sapiens* (UniProt, 2018) proteomes using Andromeda^55^ search engine. TMT based experiments in MaxQuant were performed using the ‘reporter ion MS2′ built-in quantification algorithm with reporter mass tolerance set to 0.003 Da. Cysteine carbamidomethylation was selected as a fixed modification. Methionine oxidation, acetylation of protein N-terminus, deamidation (NQ) and phosphorylation (S, T, Y) were selected as variable modifications. The enzyme specificity was set to trypsin with a maximum of 2 missed cleavages. The precursor mass tolerance was set to 20 ppm for the first search (used for mass re-calibration) and to 4.5 ppm for the main search. The datasets were filtered on posterior error probability to achieve a 1% false discovery rate on protein, peptide and site level. ‘Match between runs’ option was enabled for fractionated samples (time window 0.7 min) and “Unique and razor peptides” mode was selected to allow identification and quantification of proteins in groups (razor peptides are uniquely assigned to protein groups and not to individual proteins). Data were further analyzed as described in the Results section and in the Supplemental Dataset S2 using Microsoft Office Excel 2016 and Perseus^56^ (version 1.5.0.9).

## Supplementary information

Supplementary Figure

Supplementary dataset S1

Supplementary dataset S2
