# Supplementary material for: A plasma membrane localized protein phosphatase in Toxoplasma gondii, PPM5C, regulates attachment to host cells
Source: Sci Rep. 2019 Apr 11;9:5924. doi: 10.1038/s41598-019-42441-1 (PMC6459975; doi:10.1038/s41598-019-42441-1)
Supplement: Supplementary file 1 — Supplementary Figure [file 41598_2019_42441_MOESM1_ESM.pdf]

A plasma membrane localized protein phosphatase in *Toxoplasma gondii*, PPM5C, regulates attachment to host cells

Chunlin Yang[1], Malgorzata Broncel[3], Caia Dominicus[3], Emily Sampson[1], William J. Blakely[1], Moritz Treeck[3], Gustavo Arrizabalaga[1, 2 *]

Supplementary information

Supplemental figure S1

PPM2A(HA)

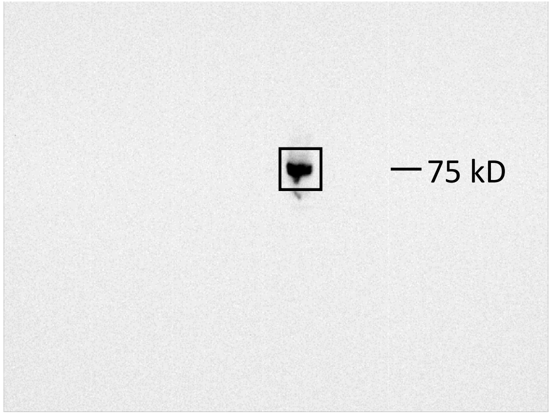

PPM5C(HA)

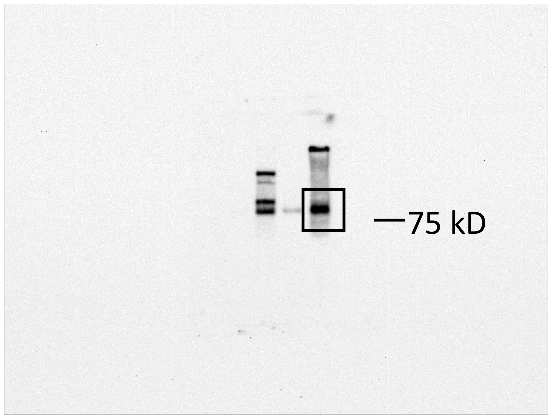

PPM2B(HA)

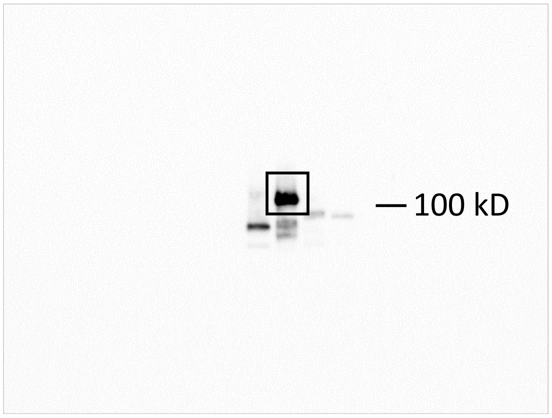

PPM11C(HA)

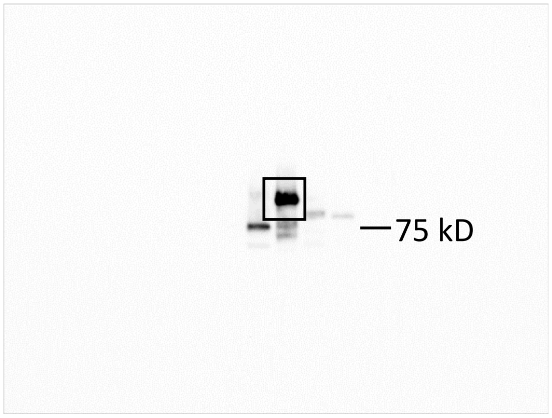

PPM3D(HA)

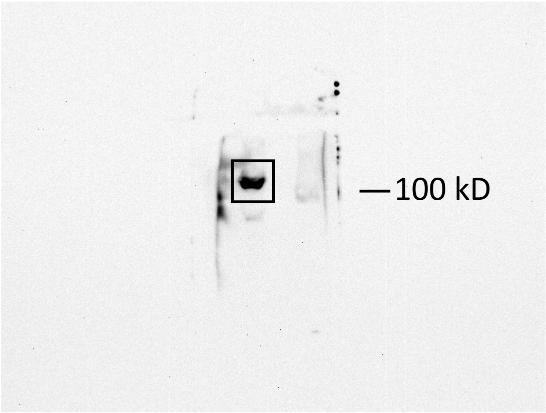

Supplemental figure S1. Original full length blots for Figure 2. Boxes indicate area shown in figure

Supplemental figure S2

+tubPPM5C(HA)

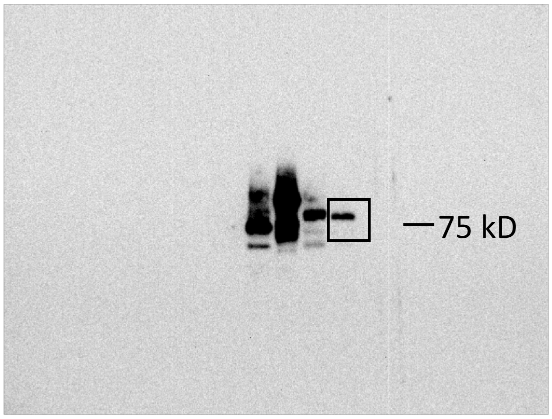

+tubPPM5C(HA)
G2A

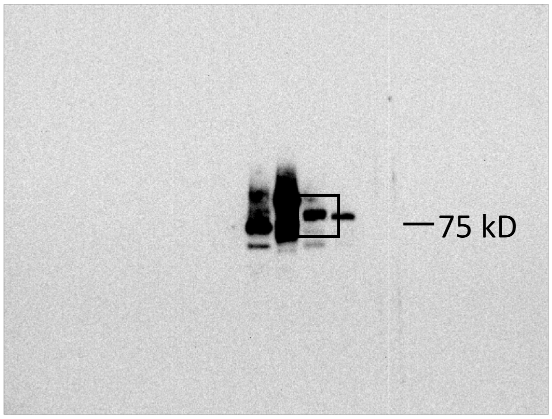

+tubPPM5C(HA)
C4A

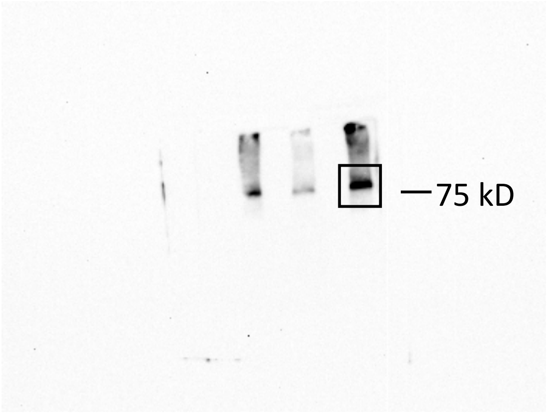

Supplemental figure S2. Original uncropped blots for Figure 3. Boxes mark areas shown in figure.

Supplemental figure S3

A.

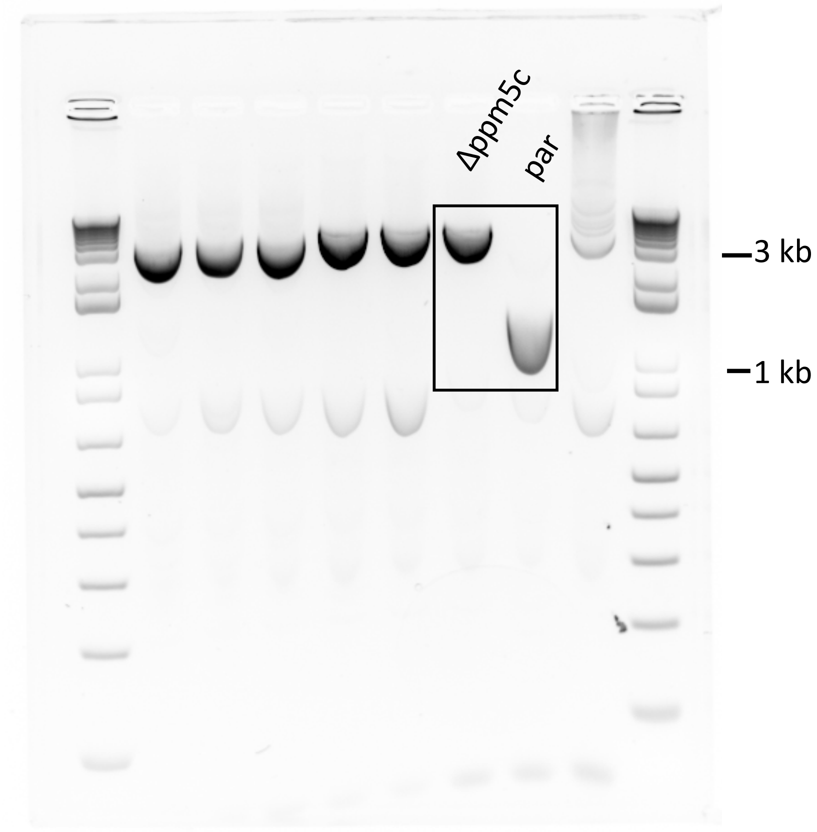

B.

Δ*ppm5c*.cp

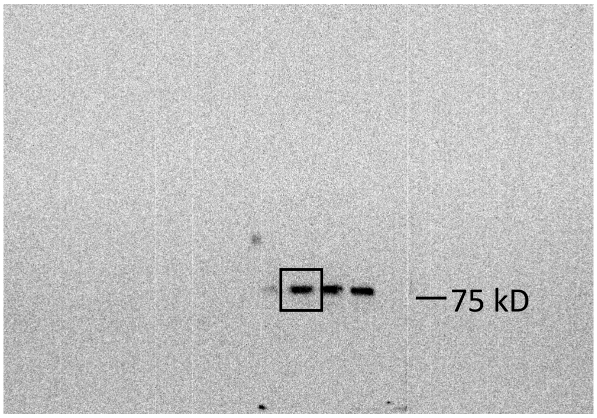

Supplemental figure S3. Original blots for westerns shown in figures 3B (A) and D (B). Boxes mark areas shown in figure.

Supplemental figure S4

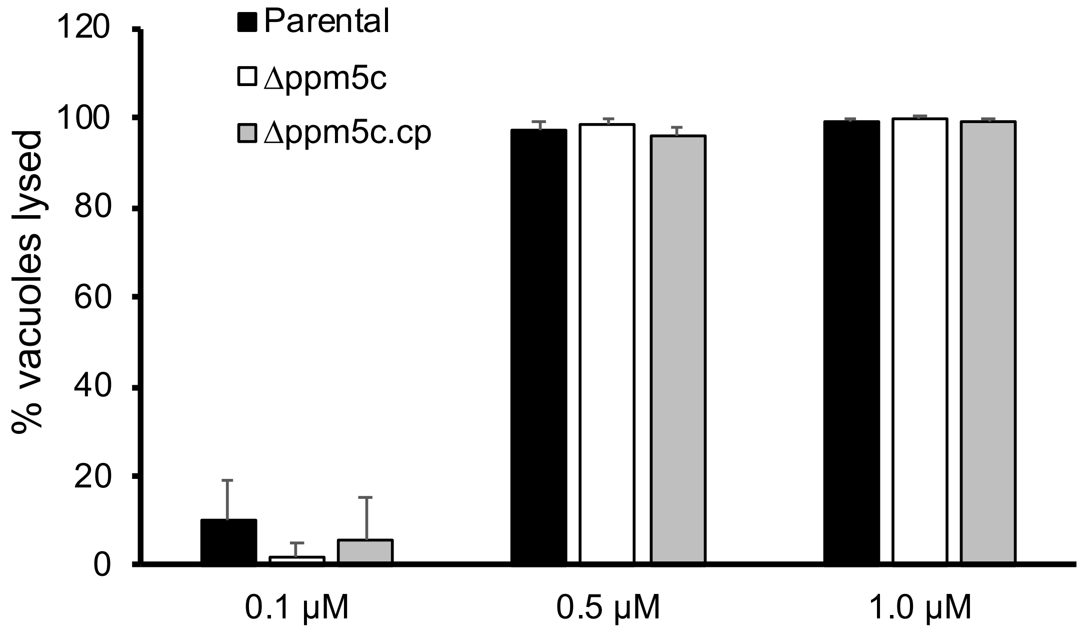

Supplemental figure S4. Parental, knockout (Δppm5c), and complemented (Δppm5c.cp) parasites were exposed to 0.1, 0.5 and 1.0 µM A23184 for two minutes. Percentage vacuoles lysed is based on no treatment controls. n=3, ±sd.

Supplemental figure S5

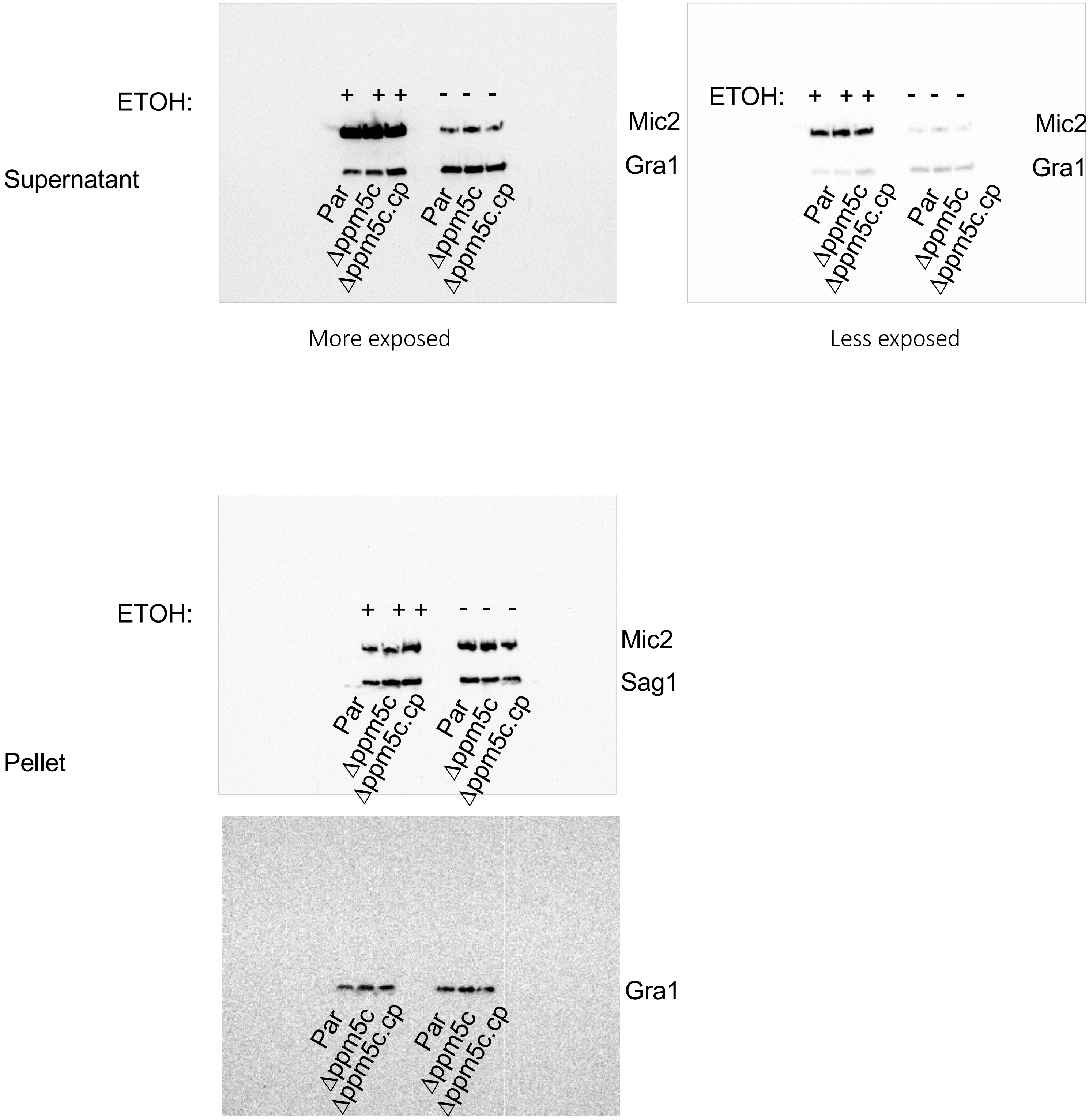

Supplemental figure S5. Originals for all the western blots shown in figure 6.

Supplemental figure S6

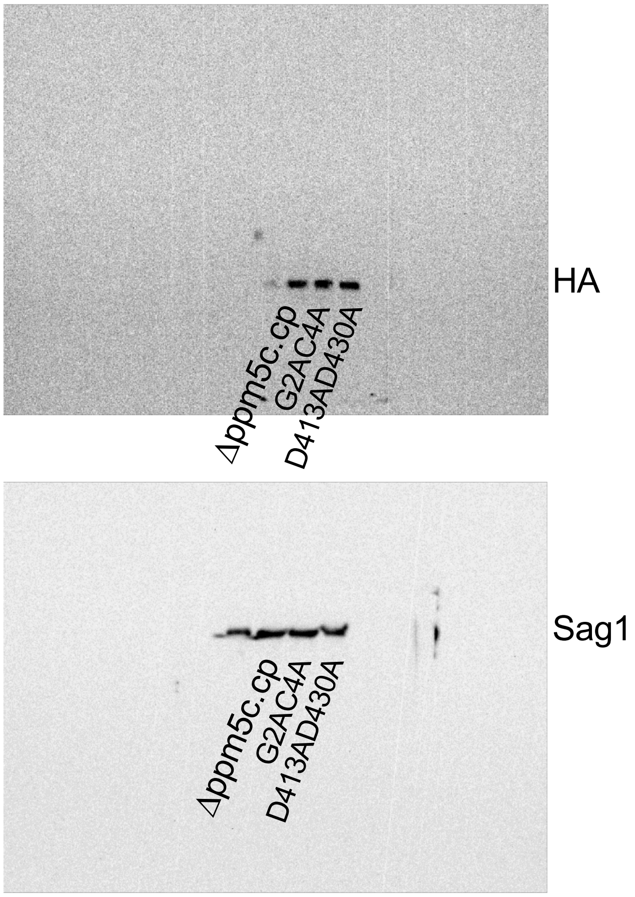

Supplemental figure S6. Originals of blots shown in figure 7.

Supplemental figure S7

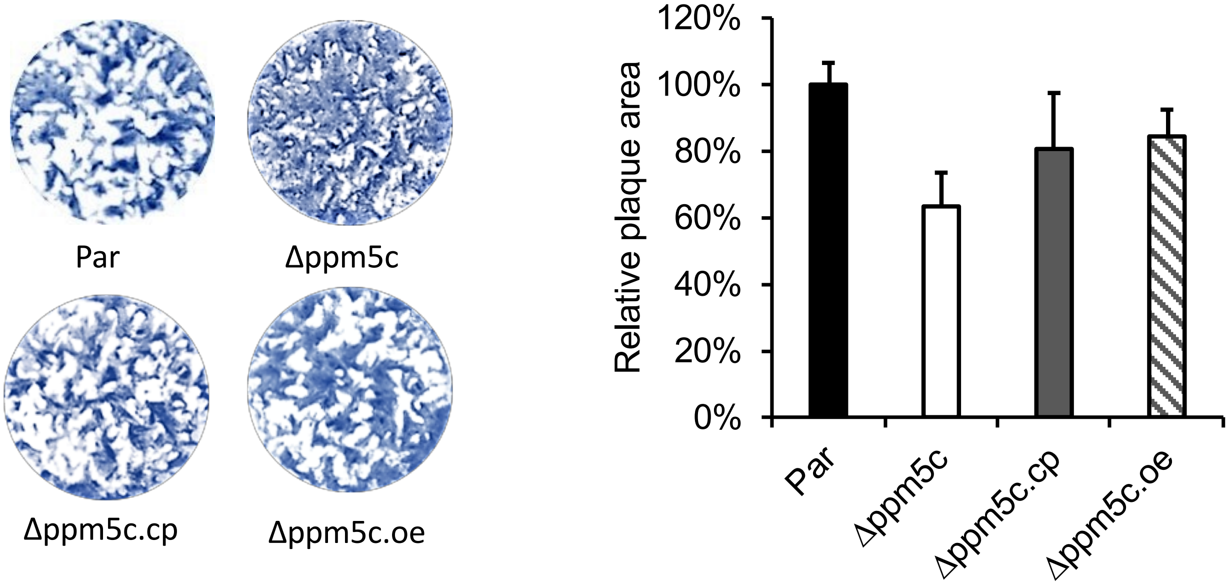

Supplemental figure S7. Plaque analysis of PPM5C knockout and complement strains. Parasites of all four strains were allowed to grow in human fibroblasts for six days before fixation and crystal violet staining to reveal plaques formed by repeating cycles of invasion, replication and egress. Representative images of plaque assays are shown as well as quantification of area cleared by plaques relative to parental strain.

Supplemental figure S8

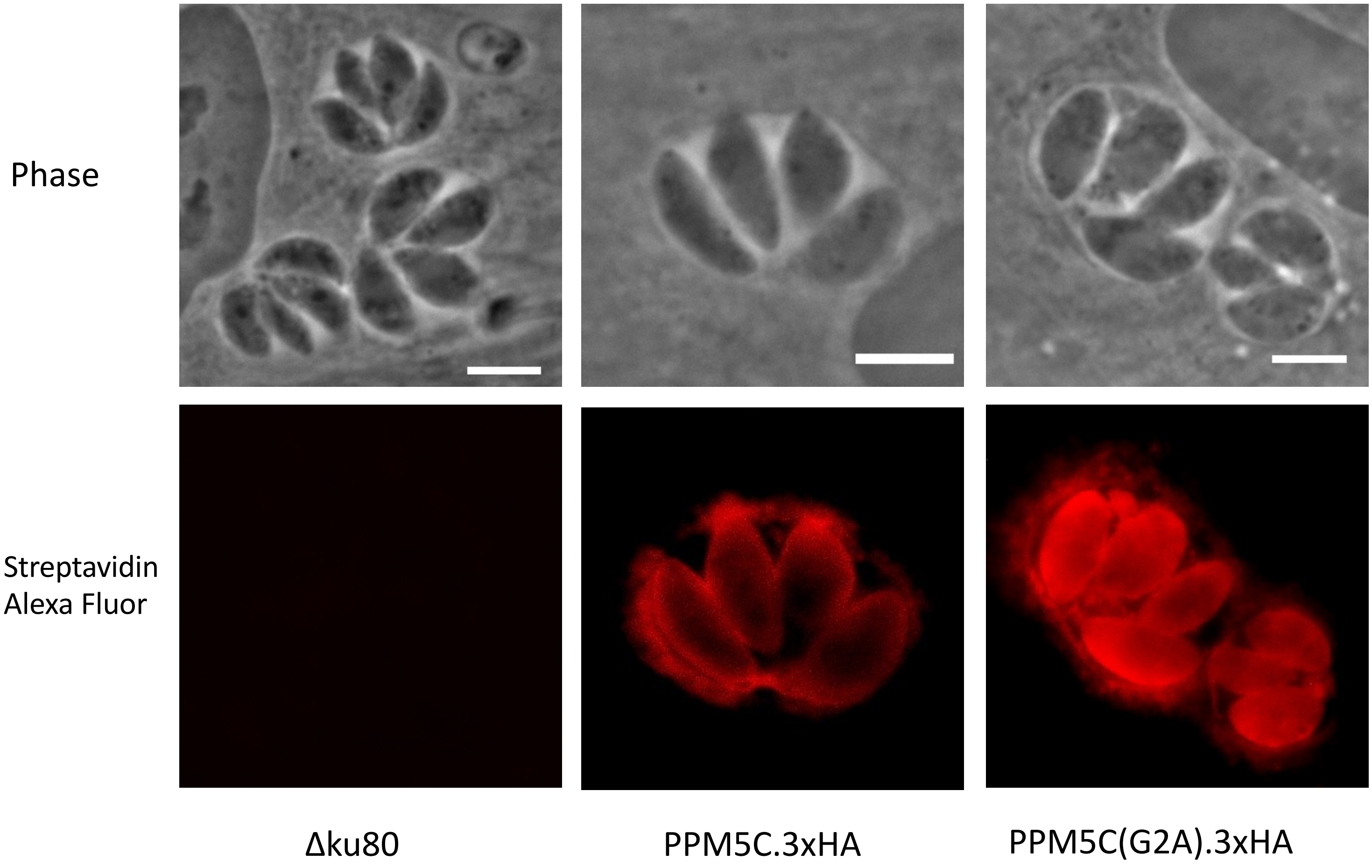

Supplemental Figure S8. Immunofluorescence of biotinylated proteins obtained by performing BAR. Biotinylation reactions were performed by following the BAR method described in the Methods section. After biotinylation, Streptavidin Alexa Fluor 594 (1:10000 dilution) was used to stain biotinylated proteins. Scale Bar: 5 ul.

Supplementary table S1. Primers used in this study.

Purpose	Primer name	Sequence
Endogeneous Tagging	PPM2A.For	ttccaatccaatttaattaaagaaagtctccgcaaacgaag
	PPM2A.Rev	ccacttccaattttaattaaCTCGTCGTTGTCCGACTCG
	PPM2B.For	ttccaatccaatttaattaaGTTGTTTGTGTGTGGCTTGGTG
	PPM2B.Rev	ccacttccaattttaattaaCGCTCGGCCGTCTG
	PPM3D.For	ttccaatccaatttaattaacttgtctctgtgtgtctctccact
	PPM3D.Rev	ccacttccaattttaattaaGTAGAGAGCGACGTTCTTCTTC
	PPM5C.For	ttccaatccaatttaattaattccctttcttcccctcctatctcg
	PPM5C.Rev	ccacttccaattttaattaaACGGAGGCAAATGATTTGACAGG
Overexpression of PPM5C	PPM5C.OE.For	TTTCGACAAAccatggGTGCATGCAAGAGCA
	PPM5C.OE.Rev	ttaattaaACGGAGGCAAATGATTTGACAG
	OE.G2A.For	CAAACCATGGcTGCATGCAAG
	OE.G2A.Rev	TCGAAAAAGGGAATTCAAG
	OE.C4A.For	CATGGGTGCAgcCAAGAGCAAG
	OE.C4A.Rev	GTTTGTCGAAAAAGGGAATTC
PPM5C knockout	PPM5C.sgRNA.For	AAGTTGCCTCCGCCTCATCGCGAGTG
	PPM5C.sgRNA.Rev	AAAACACTCGCGATGAGGCGGAGGCA
	DHFR.For	CTTCCTGTCTCTTCTCCCTCATCTTCGCGTCCTTCCTCCGC GACTCACTATAGGGAGAGCGGC
	DHFR.Rev	CCGCTCCTCCCCCCCCGCGCAAAGCCTCCGCCTCATCGCGAAGAA CATCGATTTTCCATGGCAG
	P1 (KO.test.For)	CCTTTCTGCCGACTGTTGTT
	P2 (KO.test.Rev)	TCACTGACTGCCCAGGTGTA
Complement	PPM5C.pr.CP.For	GCGAATTGGGTACCGGGCCCTTGAGATAAAGGGTGCAAACATCCG
	PPM5C.pr.CP.Rev	TTGCATGCACccatggCTCGCGGAGGAAGGACGC
	G2AC4A.For	gcagcCAAGAGCAAGGCAGCGAAG
	G2AC4A.Rev	agccatGGCTCGCGGAGGAAGGAC
	D413AD430A.For	gccttccgcctcttcggcgtcttcgctGGGCATGGGCCGAGCGGG
	D413AD430A.Rev	gctgcagcagagagccgcaaagtcggcTTGGTTCGGACTGTCTGGCTTCAGTC C
